# Glycolysis Induces Abnormal Transcription Through Histone Lactylation in T-cell Acute Lymphoblastic Leukemia

**DOI:** 10.1093/gpbjnl/qzaf029

**Published:** 2025-04-07

**Authors:** Wenyan Wu, Jingyi Zhang, Huiying Sun, Xiaoyu Wu, Han Wang, Bowen Cui, Shuang Zhao, Kefei Wu, Yanjun Pan, Rongrong Fan, Ying Zhong, Xiang Wang, Ying Wang, Xiaoxiao Chen, Jianan Rao, Ronghua Wang, Kai Luo, Xinrong Liu, Liang Zheng, Shuhong Shen, Meng Yin, Yangyang Xie, Yu Liu

**Affiliations:** Key Laboratory of Pediatric Hematology & Oncology Ministry of Health, Department of Hematology & Oncology, Pediatric Translational Medicine Institute, Shanghai Children’s Medical Center, School of Medicine, Shanghai Jiao Tong University, Shanghai 200127, China; Department of Cardiothoracic Surgery, Shanghai Children’s Medical Center, School of Medicine, Shanghai Jiao Tong University, Shanghai 200127, China; Key Laboratory of Pediatric Hematology & Oncology Ministry of Health, Department of Hematology & Oncology, Pediatric Translational Medicine Institute, Shanghai Children’s Medical Center, School of Medicine, Shanghai Jiao Tong University, Shanghai 200127, China; Key Laboratory of Pediatric Hematology & Oncology Ministry of Health, Department of Hematology & Oncology, Pediatric Translational Medicine Institute, Shanghai Children’s Medical Center, School of Medicine, Shanghai Jiao Tong University, Shanghai 200127, China; Key Laboratory of Pediatric Hematology & Oncology Ministry of Health, Department of Hematology & Oncology, Pediatric Translational Medicine Institute, Shanghai Children’s Medical Center, School of Medicine, Shanghai Jiao Tong University, Shanghai 200127, China; Key Laboratory of Pediatric Hematology & Oncology Ministry of Health, Department of Hematology & Oncology, Pediatric Translational Medicine Institute, Shanghai Children’s Medical Center, School of Medicine, Shanghai Jiao Tong University, Shanghai 200127, China; Key Laboratory of Pediatric Hematology & Oncology Ministry of Health, Department of Hematology & Oncology, Pediatric Translational Medicine Institute, Shanghai Children’s Medical Center, School of Medicine, Shanghai Jiao Tong University, Shanghai 200127, China; Key Laboratory of Pediatric Hematology & Oncology Ministry of Health, Department of Hematology & Oncology, Pediatric Translational Medicine Institute, Shanghai Children’s Medical Center, School of Medicine, Shanghai Jiao Tong University, Shanghai 200127, China; Department of Cardiothoracic Surgery, Shanghai Children’s Medical Center, School of Medicine, Shanghai Jiao Tong University, Shanghai 200127, China; Key Laboratory of Pediatric Hematology & Oncology Ministry of Health, Department of Hematology & Oncology, Pediatric Translational Medicine Institute, Shanghai Children’s Medical Center, School of Medicine, Shanghai Jiao Tong University, Shanghai 200127, China; Key Laboratory of Pediatric Hematology & Oncology Ministry of Health, Department of Hematology & Oncology, Pediatric Translational Medicine Institute, Shanghai Children’s Medical Center, School of Medicine, Shanghai Jiao Tong University, Shanghai 200127, China; Key Laboratory of Pediatric Hematology & Oncology Ministry of Health, Department of Hematology & Oncology, Pediatric Translational Medicine Institute, Shanghai Children’s Medical Center, School of Medicine, Shanghai Jiao Tong University, Shanghai 200127, China; Key Laboratory of Pediatric Hematology & Oncology Ministry of Health, Department of Hematology & Oncology, Pediatric Translational Medicine Institute, Shanghai Children’s Medical Center, School of Medicine, Shanghai Jiao Tong University, Shanghai 200127, China; Key Laboratory of Pediatric Hematology & Oncology Ministry of Health, Department of Hematology & Oncology, Pediatric Translational Medicine Institute, Shanghai Children’s Medical Center, School of Medicine, Shanghai Jiao Tong University, Shanghai 200127, China; Key Laboratory of Pediatric Hematology & Oncology Ministry of Health, Department of Hematology & Oncology, Pediatric Translational Medicine Institute, Shanghai Children’s Medical Center, School of Medicine, Shanghai Jiao Tong University, Shanghai 200127, China; Key Laboratory of Pediatric Hematology & Oncology Ministry of Health, Department of Hematology & Oncology, Pediatric Translational Medicine Institute, Shanghai Children’s Medical Center, School of Medicine, Shanghai Jiao Tong University, Shanghai 200127, China; Department of Cardiothoracic Surgery, Shanghai Children’s Medical Center, School of Medicine, Shanghai Jiao Tong University, Shanghai 200127, China; Department of Cardiothoracic Surgery, Shanghai Children’s Medical Center, School of Medicine, Shanghai Jiao Tong University, Shanghai 200127, China; Key Laboratory of Pediatric Hematology & Oncology Ministry of Health, Department of Hematology & Oncology, Pediatric Translational Medicine Institute, Shanghai Children’s Medical Center, School of Medicine, Shanghai Jiao Tong University, Shanghai 200127, China; Key Laboratory of Pediatric Hematology & Oncology Ministry of Health, Department of Hematology & Oncology, Pediatric Translational Medicine Institute, Shanghai Children’s Medical Center, School of Medicine, Shanghai Jiao Tong University, Shanghai 200127, China; Department of Cardiothoracic Surgery, Shanghai Children’s Medical Center, School of Medicine, Shanghai Jiao Tong University, Shanghai 200127, China; Key Laboratory of Pediatric Hematology & Oncology Ministry of Health, Department of Hematology & Oncology, Pediatric Translational Medicine Institute, Shanghai Children’s Medical Center, School of Medicine, Shanghai Jiao Tong University, Shanghai 200127, China; Key Laboratory of Pediatric Hematology & Oncology Ministry of Health, Department of Hematology & Oncology, Pediatric Translational Medicine Institute, Shanghai Children’s Medical Center, School of Medicine, Shanghai Jiao Tong University, Shanghai 200127, China; Fujian Children’s Hospital, Fujian Branch of Shanghai Children’s Medical Center Affiliated to Shanghai Jiao Tong University School of Medicine, Fuzhou 350014, China

**Keywords:** Pediatric T-cell acute lymphoblastic leukemia, Histone H3 lysine 18 lactylation, Warburg effect, Transcription dysregulation, Metabolism epigenetic interplay

## Abstract

The Warburg effect, characterized by excessive lactate production, and transcriptional dysregulation are two hallmarks of tumors. However, the precise influence of lactate on epigenetic modifications at a genome-wide level and its impact on gene transcription in tumor cells remain unclear. In this study, we conducted genome-wide profiling of histone H3 lysine 18 lactylation (H3K18la) in T-cell acute lymphoblastic leukemia (T-ALL). We observed elevated lactate and H3K18la levels in T-ALL cells compared to normal T cells, with H3K18la levels positively associated with cell proliferation. Accordingly, we observed a significant shift in genome-wide H3K18la modifications from T cell immunity in normal T cells to leukemogenesis in T-ALL, correlated with altered gene transcription profiles. We showed that H3K18la primarily functions in active transcriptional regulation and observed clusters of H3K18la modifications resembling super-enhancers. Disrupting H3K18la modification revealed both synergistic and divergent changes between H3K18la and histone H3 lysine 27 acetylation (H3K27ac) modifications. Finally, we found that the high transcription of H3K18la target genes, *IGFBP2* and *IARS*, is associated with inferior prognosis of T-ALL. These findings enhance our understanding of how metabolic disruptions contribute to transcription dysregulation through epigenetic changes in T-ALL, underscoring the interplay of histone modifications in maintaining oncogenic epigenetic stability.

## Graphical abstract

**Graphical Abstract:**
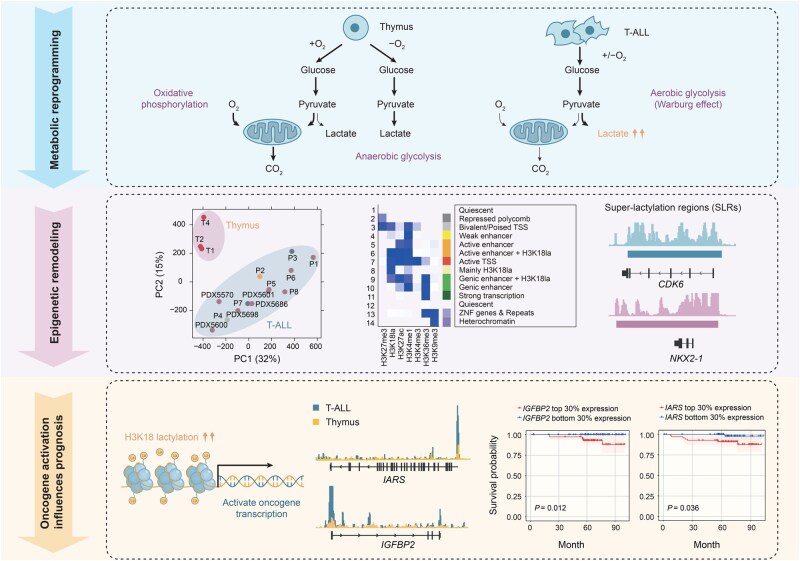

## Introduction

Metabolic reprogramming stands as a hallmark of cancer, characterized by cells preferring glycolysis over oxidative phosphorylation for energy production, even in oxygen-rich environments. This is a phenomenon known as the Warburg effect [1]. Lactate, a key glycolysis product, is notably increased in cancer. In recent years, lactate has garnered attention not only as a glycolysis by-product but also as a pivotal signaling molecule and metabolic intermediary, crucial to cellular function [2,3]. Advancements in high-resolution liquid chromatography-tandem mass spectrometry (LC-MS/MS) have identified histone lysine lactylation (Kla) as a novel epigenetic mark [4]. Investigations utilizing cell-free systems with recombinant chromatin have shown that Kla of histones can directly stimulate gene transcription [4], revealing another connection between metabolism and epigenetic regulation. This is particularly relevant to cancer, as both metabolic and epigenetic dysregulation play significant roles in carcinogenesis. Subsequent studies have elucidated the role of histone lactylation in cancer, including its promotion of tumor cell proliferation, migration, and drug resistance [5–8]. Notably, histone lactylation can also modulate immune cells within the tumor microenvironment, contributing to immune evasion [9–11]. Among the various sites of histone Kla, lactylation at histone H3 lysine 18 (H3K18la) has received considerable attention. Research has shown that H3K18la marks promoters and active enhancers with a tissue-specific distribution pattern similar to that of histone H3 lysine 27 acetylation (H3K27ac) [12], and is associated with chromatin accessibility [13]. This accumulating evidence underscores the connection between metabolism and transcriptional regulation in tumor cells through epigenetic changes.

T-cell acute lymphoblastic leukemia (T-ALL) arises from a blockade in the early stages of thymic cell differentiation, exhibiting significant transcriptional and epigenetic dysregulation [14–16]. Studies have identified genomic aberrations that are responsible for constitutive activation of key transcription factors (such as TAL1/2, LMO1/2, and LYL1) involved in T cell differentiation as central drivers to T-ALL pathogenesis [16,17]. Furthermore, mutations in essential epigenetic factor genes are prevalent in approximately 68% of pediatric T-ALL cases, including mutations in *EZH2*, *SUZ12*, and *WT1*, among others. These mutations constitute one of the most frequently mutated pathways in this malignancy and are associated with a poorer prognosis for T-ALL patients [16,18–20]. In addition to genomic and epigenetic aberrations, recent studies have revealed metabolic dysregulation in various cancer types, including leukemia [21–24]. Dysfunctions in aerobic glycolysis, glutamine catabolism, and macromolecular synthesis, among other processes, are commonly observed in tumors. It is increasingly evident that metabolism interacts with epigenetic modifications to play a crucial role in tumorigenesis and treatment response. However, the intricate mechanisms linking cellular metabolism with the epigenetic and transcriptional regulatory frameworks in T-ALL remain elusive, particularly concerning the recently identified histone Kla.

In this study, we characterized genome-wide H3K18la modification in pediatric T-ALL samples. We systematically compared H3K18la with other key histone modification marks of established functions to understand its potential role alongside the current epigenetic landscape of T-ALL. Through comparative analysis with normal T cells, we unveiled that H3K18la in T-ALL is notably augmented in regions governing oncogenic transcription factors and forms clustered modifications in the genome, resembling super-enhancers (SEs). We further explored the transcription levels of H3K18la target genes and their correlation with patient prognosis to evaluate the clinical potential of H3K18la in stratifying high-risk T-ALL. Additionally, we perturbed H3K18la modification by targeting lactate dehydrogenase (LDH) to validate its role in transcriptional regulation and investigate its interplay with H3K27ac modification at these regions.

## Results

### Elevated genome-wide H3K18la modifications in T-ALL

Tumor cells typically operate within a state of heightened glycolytic metabolism, which is crucial for the initiation and progression of malignancies, accompanied by increased lactate production. To understand the role of histone lactylation modifications in the epigenetic remodeling of T-ALL, we compared the genome-wide H3K18la profiles between normal thymus tissues representing normal T cells and T-ALL tumor samples. We conducted H3K18la and H3K27ac chromatin immunoprecipitation sequencing (ChIP-seq), alongside RNA sequencing (RNA-seq) for 13 T-ALL samples, comprising 8 primary tumor samples collected at diagnosis and 5 patient-derived xenograft (PDX) samples. Additionally, we performed H3K18la ChIP-seq in 3 normal pediatric thymus tissues (Table S1). The molecular subtypes of T-ALL samples were determined by analyzing the RNA-seq data (see Materials and methods).

**Figure 1:**
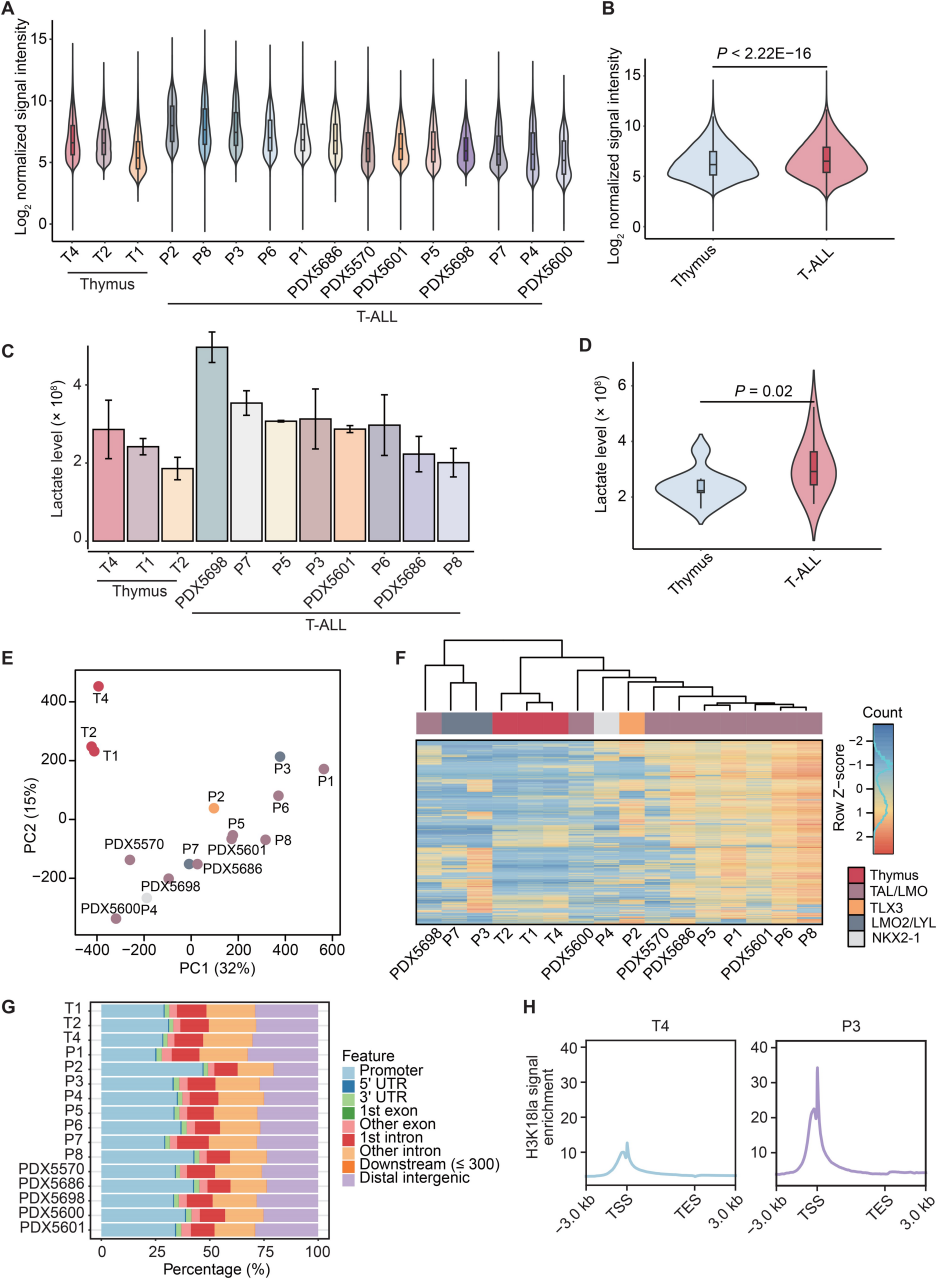
Histone H3K18la modification in normal T cells and pediatric T-ALL tumor cells **A**. Violin plot showing the normalized signal intensity of H3K18la peaks from ChIP-seq data in normal thymus tissues (*n* = 3) and primary/PDX T-ALL samples (*n* = 13). Data are downsampled before comparison. **B**. Violin plot depicting the difference in normalized signal intensity of H3K18la peaks from ChIP-seq data between normal thymus tissues and T-ALL samples. Statistical significance was determined by the Wilcoxon signed-rank test. Data are downsampled before comparison. **C**. Histogram illustrating intracellular lactate levels in normal thymus tissues (*n* = 3) and primary/PDX T-ALL samples (*n* = 8). Data are represented by mean ± SD. **D**. Violin plot showing the difference in lactate levels between normal thymus tissues and T-ALL samples. Statistical significance was determined by the Wilcoxon signed-rank test. **E**. PCA displaying clustering information of normalized H3K18la signals on consensus peaks between normal thymus tissues and T-ALL samples. **F**. Unsupervised clustering analysis of the top 1000 most variable H3K18la consensus peaks from T-ALL and normal thymus samples after normalization. TAL/LMO indicates the T-cell acute lymphocytic leukemia 1 (*TAL1*) and LIM domain only 1/2 (*LMO1/2*) overexpression subtype; TLX3 indicates the T-cell leukemia homeobox 3 (*TLX3*) overexpression subtype; LMO2/LYL indicates the subtype defined by the high expression of *LMO2* and lymphoblastic leukemia derived sequence 1 (*LYL1*); and NKX2-1 indicates the subtype defined by aberrant expression of NK2 homeobox 1 (*NKX2-1*). **G**. and **H**. Distribution patterns of H3K18la in the genome (G) and in gene regions (H) in representative T-ALL and thymic cells. H3K18la, histone H3 lysine 18 lactylation; T-ALL, T-cell acute lymphoblastic leukemia; ChIP-seq, chromatin immunoprecipitation sequencing; PDX, patient-derived xenograft; PCA, principal component analysis; PC, principal component; SD, standard deviation; UTR, untranslated region; TSS, transcription start site; TES, transcription end site.

We applied a standardized data processing protocol to all datasets (see Materials and methods; Table S2). Across all samples, we identified a median of 50,740.50 H3K18la peaks, ranging from 30,177 to 84,692 peaks. The median H3K18la signal spanned approximately 2.20% of the entire genomic region. We observed no significant differences in either the number of H3K18la peaks or the genomic regions that it covered between normal thymic cells and T-ALL (*P* = 0.36 and *P* = 0.19, respectively, Wilcoxon signed-rank test) (Figure S1A and B). However, our analysis revealed that the intensity of H3K18la modifications in T-ALL was significantly higher than that in normal T cells (*P* < 2.22E−16, Wilcoxon signed-rank test) (**Figure 1**A and B). Consistently, lactate levels were elevated in T-ALL compared to normal thymus (*P =* 0.02, Wilcoxon signed-rank test) (Figure 1C and D; Table S3). Principal component analysis (PCA) of H3K18la intensities clearly distinguished tumors from normal samples, with PDX tumors grouped together with primary T-ALL samples (Figure 1E), indicating a marked difference between T-ALL and normal T cells. Furthermore, unsupervised clustering analysis revealed that patients with the same T-ALL subtype shared similar H3K18la signal patterns (Figure 1F), suggesting a potential association between H3K18la profiles and genomic aberrations in T-ALL.

Next, we analyzed the genomic distribution of H3K18la-modified regions. The results revealed enrichment of H3K18la modifications around gene regions in the genome compared to distal intergenic regions (Figure 1G), with more prominent modification signals observed in gene promoters (Figure 1H, Figure S1C). Notably, the H3K18la patterns were comparable between normal T cells and T-ALL cells, consistent with previous reports in macrophage and muscle tissues [4,12].

### Activating role of H3K18la in transcriptional regulation

**Figure 2:**
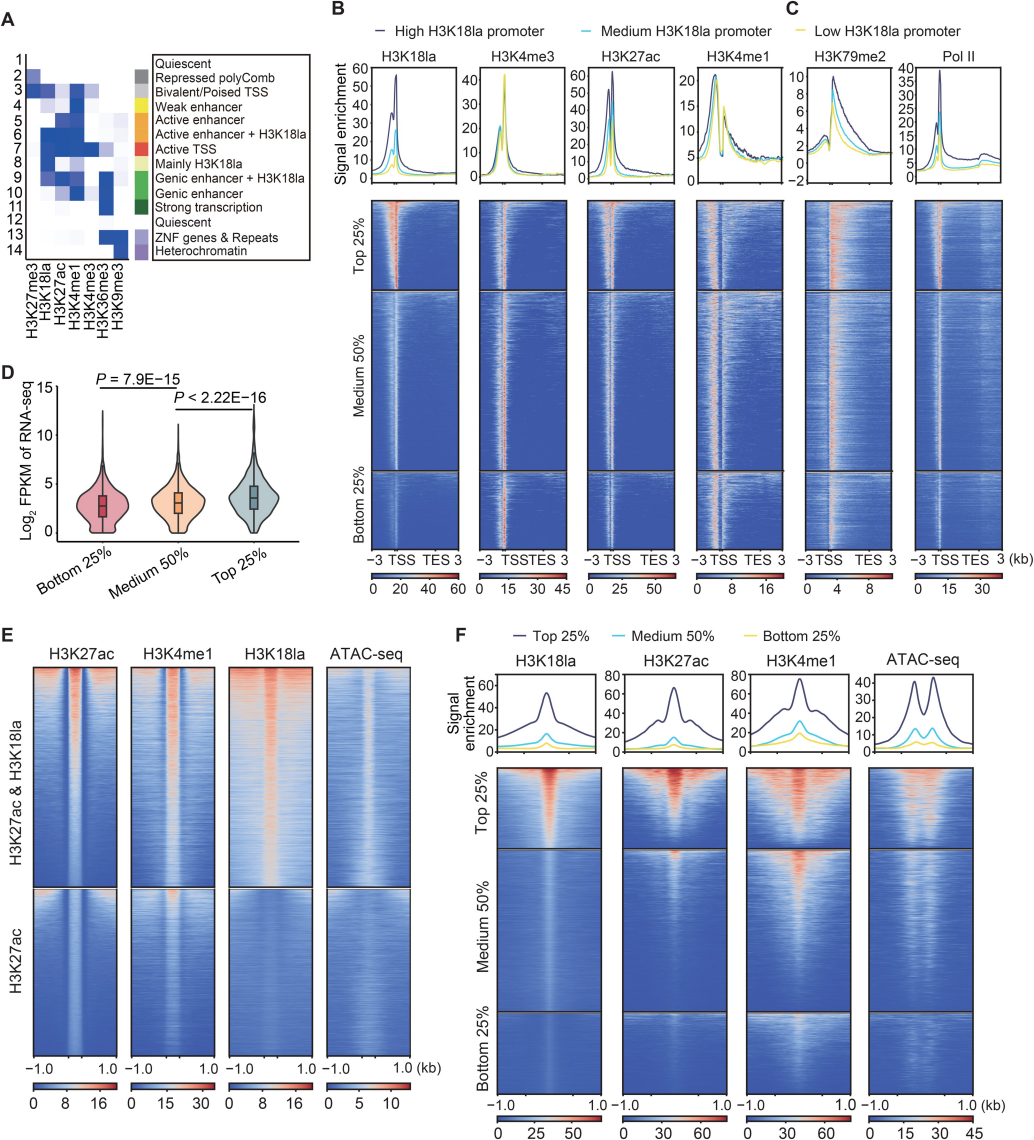
H3K18la modification was associated with active transcriptional regulation in the genome **A**. ChromHMM chromatin state analysis for the DND41 cell line. **B**. and **C**. Promoter regions inferred as state 7 in (A) are organized into groups based on the intensity of H3K18la modification. The relative signal intensities for H3K4me3 (B), H3K27ac (B), H3K4me1 (B), H3K79me2 (C), and Pol II (C) across these groups are shown. **D**. Violin plot showing transcription level of genes within the H3K18la intensity-defined groups as established in (B and C). Statistical significance was determined by the Wilcoxon signed-rank test. **E**. Plots of signal intensity for modifications of H3K27ac, H3K4me1, H3K18la, and chromatin accessibility measured by ATAC-seq in enhancer regions defined by H3K27ac. Genomic regions were separated into two groups based on the presence of H3K18la modification. **F**. Enhancer regions defined by H3K27ac are categorized by varying levels of H3K18la modification signal intensity. The plots illustrate the signal intensity for modifications of H3K18la, H3K27ac, H3K4me1, and chromatin accessibility measured by ATAC-seq within these groups. H3K4me3, histone H3 lysine 4 trimethylation; H3K27ac, histone H3 lysine 27 acetylation; H3K4me1, histone H3 lysine 4 monomethylation; H3K36me3, histone H3 lysine 36 trimethylation; H3K9me3, histone H3 lysine 9 trimethylation; H3K27me3, histone H3 lysine 27 trimethylation; H3K79me2, histone H3 lysine 79 dimethylation; RNA-seq, RNA sequencing; Pol II, RNA polymerase II; ATAC-seq, assay for transposase-accessible chromatin with high-throughput sequencing; FPKM, fragments per kilobase of transcript per million mapped reads.

To understand the interplay between H3K18la and other histone modifications, we performed H3K18la ChIP-seq experiment in the T-ALL cell line DND41 and integrated H3K18la with other key histone modifications with established functions. These included the promoter marker histone H3 lysine 4 trimethylation (H3K4me3), enhancer markers H3K27ac and histone H3 lysine 4 monomethylation (H3K4me1), the transcriptionally engaged region marker histone H3 lysine 36 trimethylation (H3K36me3), the heterochromatin region marker histone H3 lysine 9 trimethylation (H3K9me3), and the polycomb complex region marker histone H3 lysine 27 trimethylation (H3K27me3), obtained from the Encyclopedia of DNA Elements (ENCODE) project [25]. The results revealed a notable co-occurrence of H3K18la with active transcriptional regulatory regions marked by H3K4me3, H3K4me1, and H3K27ac (Figure S2A–C). Conversely, the overlap between H3K18la and repressive markers H3K9me3 and H3K27me3 was less pronounced (Figure S2D and E). By employing ChromHMM to integrate these datasets, we identified 14 distinct chromatin states (**Figure 2**A). H3K18la was predominantly involved in states 3, 6, 7, 8, and 9, with the majority of states associated with regulatory regions of active transcription, including active promoters (state 7) and active enhancers (states 6 and 9). These colocalization patterns suggest an activating role of H3K18la in gene transcription. Notably, H3K18la further subdivided these active regulatory regions, indicating its specific function in transcriptional regulation. Furthermore, we observed that state 8 was mainly contributed by H3K18la modifications. Weak H3K4me1 modifications were observed in this state, suggesting a potential weak enhancer region. Indeed, assay for transposase-accessible chromatin with high-throughput sequencing (ATAC-seq) data indicated that regions with H3K18la alone were less accessible than regions where H3K18la coexisted with other active histone modifications (Figure S2F).

To better understand the function of H3K18la in transcriptional regulation, we analyzed the interactions between H3K18la and other active histone modifications. Initially, we examined the active promoters of state 7 identified through ChromHMM. These promoters were further categorized into three subgroups based on the intensity of H3K18la modifications. We observed a positive correlation between H3K18la and other histone modifications (H3K27ac, H3K4me1, and H3K4me3) in these regions (*P* < 2.2E−16 for all tests, Kruskal–Wallis test) (Figure 2B). Additionally, a significant correlation was found between H3K18la and histone H3 lysine 79 dimethylation (H3K79me2), which is closely related to transcription elongation [26,27]. Consistently, we noted a strong association between H3K18la and RNA polymerase II (Pol II) occupancy (Figure 2C). The profiles of these histone modifications and Pol II in promoters and gene bodies integratively presented a dynamic landscape across the gene regions, revealing a significant association of increased H3K18la intensity with both high promoter activity and transcriptional activity [28]. This was further supported by the significant positive association observed between H3K18la levels at promoters and gene transcription levels detected from RNA-seq (Figure 2D).

We further investigated the association of H3K18la with active enhancers. To delineate enhancer regions for our analysis, we included areas marked by H3K27ac and explicitly excluded regions identified as promoters. Within these specified enhancer regions, over half displayed H3K18la signals. Remarkably, enhancers marked by H3K18la not only exhibited stronger signals of H3K27ac but also demonstrated greater chromatin accessibility (Figure 2E). Consistent results were observed when defining active enhancers using H3K4me1 (Figure S2G). Further subdivision of the H3K18la-marked enhancers based on signal intensity revealed a significant proportional relationship between the intensity of H3K18la signals and that of other active enhancer markers, as well as chromatin accessibility (*P* < 2.2E−16 for all tests, Kruskal–Wallis test) (Figure 2F, Figure S2H).

We observed similar results when analyzing H3K18la and H3K27ac modifications in T-ALL tumor samples. The majority of H3K27ac sites were accompanied by H3K18la signals, and the intensity of H3K27ac signals was directly proportional to H3K18la levels at promoters (Figure S3) and enhancer regions (Figure S4). Genes with promoters co-marked by both H3K18la and H3K27ac exhibited higher expression than those with promoters marked only by H3K27ac or H3K18la (Figure S5). This proportionality underscores the regulatory role of H3K18la in active transcriptional regulation, in conjunction with classical enhancer-related modifications, and suggests its involvement in chromatin opening.

### Super-lactylation regions delineated genes with essential functions

**Figure 3:**
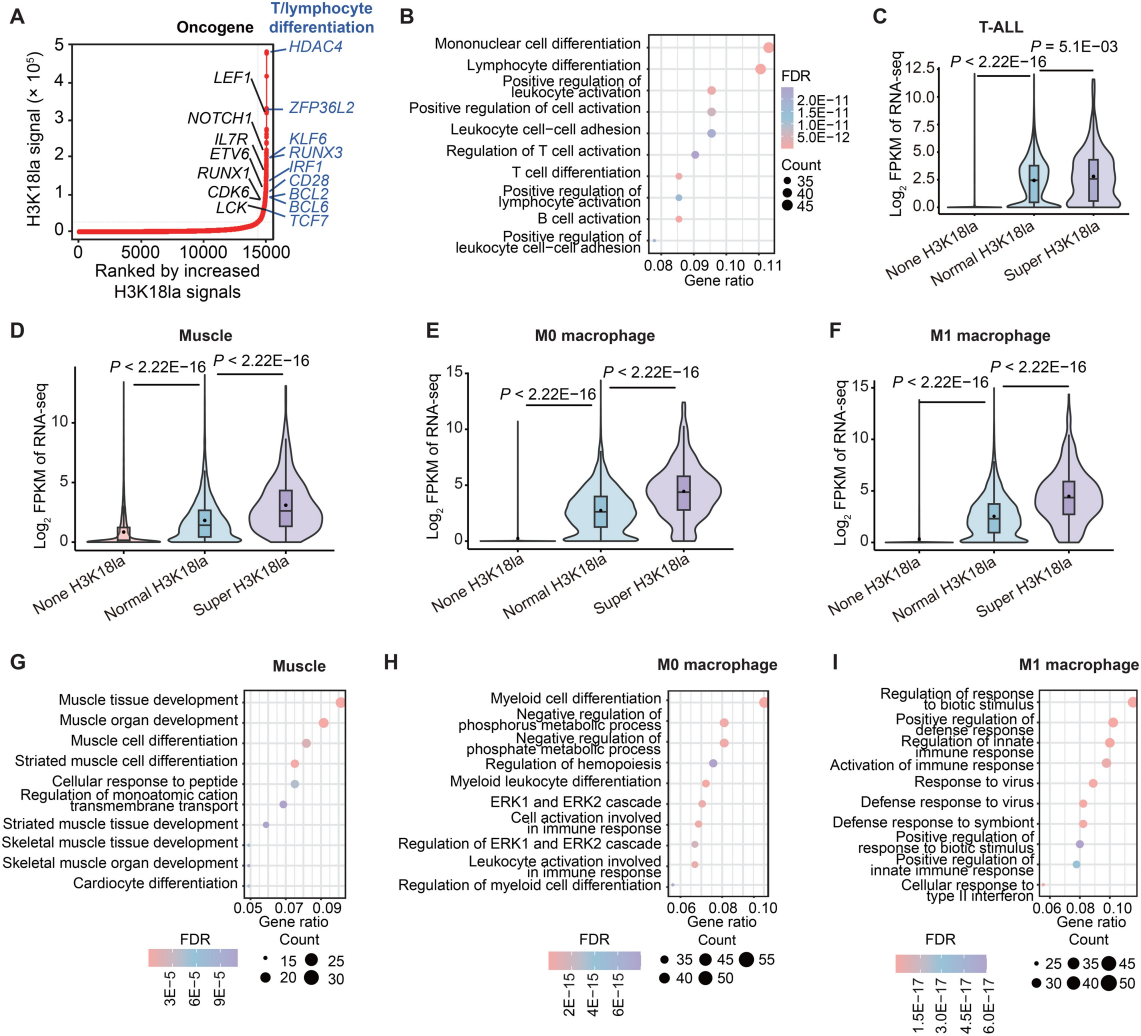
SLRs in the genome of normal and tumor cells **A**. Distribution of H3K18la signals in DND41 cells. H3K18la signals within a 12.5-kb region were clustered. Potential oncogenes in T-ALL from the COSMIC database were labeled on the plot, together with the genes implicated in T/lymphocyte differentiation as defined by the GO database. **B**. Gene set enrichment analysis displays pathways enriched in genes associated with SLRs in DND41 cells, listing the top 10 pathways with the most significant FDR. **C**.–**F**. Violin plots illustrate the transcriptional differences among genes associated with different H3K18la signals in T-ALL (C) as well as in normal muscle tissue (D), M0 macrophage (E), and M1 macrophage (F). Genes were categorized into three groups, including no H3K18la signal (none H3K18la), normal H3K18la signals (normal H3K18la), and H3K18la signals associated with SLRs (super H3K18la). Wilcoxon signed-rank test was performed for each comparison. **G**.–**I**. Gene set enrichment analysis shows pathways enriched in genes associated with SLRs in different tissue types, including muscle tissue (G), M0 macrophage (H), and M1 macrophage (I). The top 10 most significantly enriched pathways are shown. COSMIC, Catalogue of Somatic Mutations in Cancer; GO, Gene Ontology; SLR, super-lactylation region; FDR, false discovery rate.

Within the H3K18la signal landscape, we observed genomic regions with clusters of pronounced signal density resembling SEs [29]. By applying the Rank Ordering of Super-Enhancers (ROSE) algorithm [30] to H3K18la ChIP-seq data collected from T-ALL cell line DND41, we identified zones of pronounced H3K18la (see Materials and methods), exhibiting significantly more intense modifications compared to other regions. These regions were designated as “super-lactylation regions (SLRs)”. Notably, SLRs marked several oncogenes involved in T-ALL pathogenesis, such as *NOTCH1*, *CDK6*, and *IL7R*, as well as key transcription factor genes essential for T-cell differentiation, including *KLF6*, *RUNX3*, and *BCL2* (**Figure 3**A). Gene Ontology (GO) enrichment analysis revealed substantial enrichment of SLR-associated genes in T-cell and lymphocyte differentiation pathways (Figure 3B), with these genes exhibiting higher transcription levels than those associated with normal histone lactylation (*P* = 5.1E−03, Wilcoxon signed-rank test) (Figure 3C).

Next, we compared SLRs to SEs across the genome. Firstly, a discernible overlap was observed between genes associated with SLRs and SEs (Figure S6A and B). The overlapping sectors were significantly represented within the lymphocyte developmental pathways (Figure S6C), indicating a concerted regulation by these signals of genes central to cellular functionality. Consistently, the SLRs were detected not only in tumors but also in normal thymus tissues (where they were associated with genes implicated in lymphocyte development) (Figures S6D and S7).

To further validate the role of SLRs in regulating the transcription of genes related to cell identity and function, we expanded our analysis to different tissue types, including human muscle and mouse macrophage cells, using published H3K18la ChIP-seq datasets [4,12]. We identified SLRs present in both tissue types, associated with significantly robust expression across all examined contexts (*P* < 2.2E−16 for all tests, Wilcoxon signed-rank test) (Figure 3D–F), confirming SLRs as a general phenomenon in various cell types.

Subsequently, we confirmed that genes associated with SLRs reflected lineage specificity. For instance, in muscle tissue, SLR-associated genes were preferentially involved in muscle development and functional pathways (Figure 3G). In M0 macrophages, these genes were significantly enriched in myeloid lineage differentiation, whereas in M1 macrophages, they shifted toward immunological response pathways (Figure 3H and I). These results suggest that SLRs function in concert with SEs in regulating the transcription of genes that define cellular identity and function and are characteristically associated with augmented transcriptional activity.

### H3K18la profiles in T-ALL samples shifted to the regulation of genes with key pathogenetic functions

**Figure 4:**
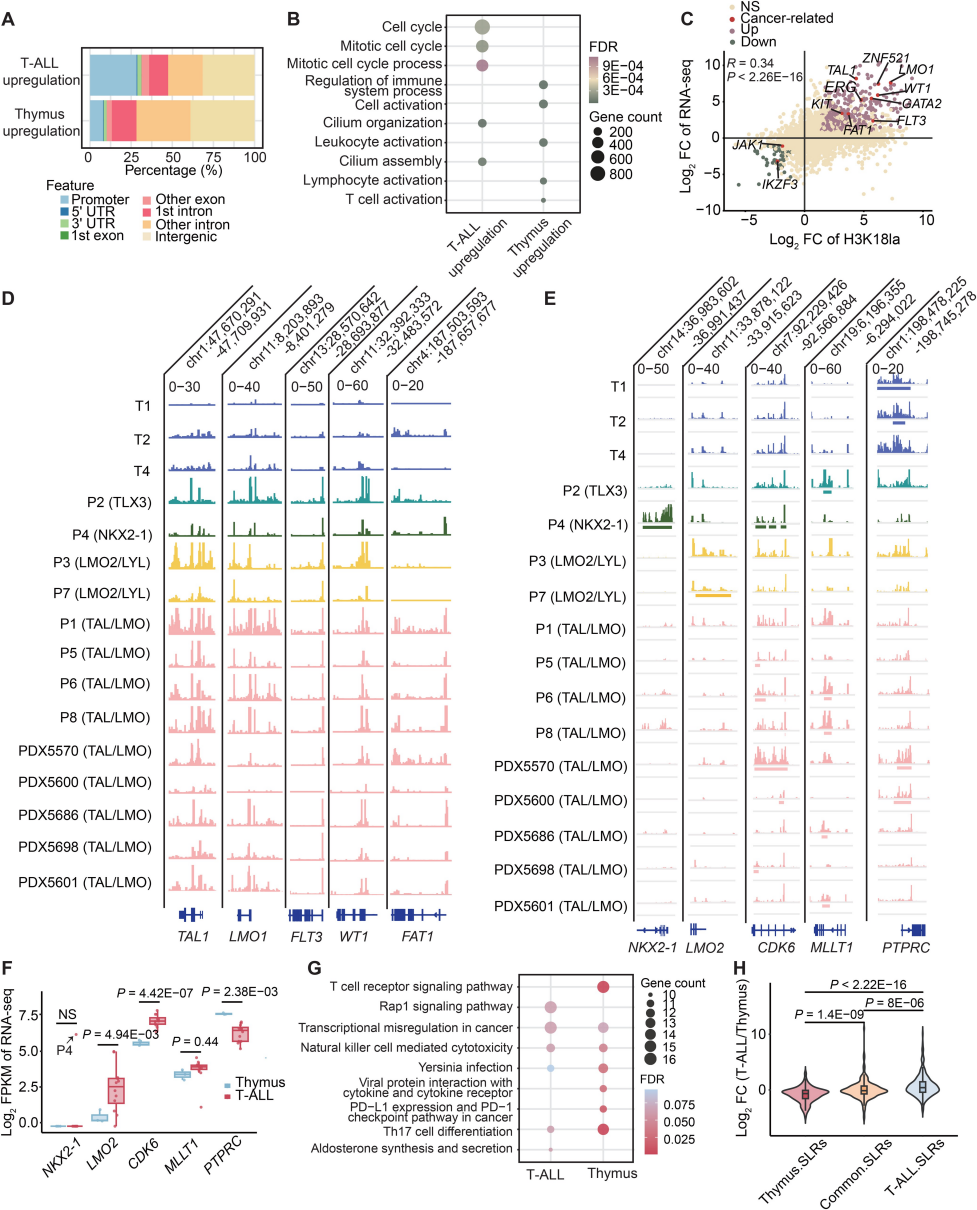
Differential H3K18la modification profiles between T-ALL tumor cells and normal T cells **A**. Genomic distribution of H3K18la-modified regions upregulated in T-ALL or thymus. **B**. Gene set enrichment analysis comparing functional categories enriched in genes associated with upregulated H3K18la modifications in T-ALL or thymus. The top 5 functional categories with the most significant FDR in T-ALL or thymus are shown. **C**. Scatter plot illustrates the correlation between changes in H3K18la signal intensity in promoters (log_2_ FC of H3K18la ChIP-seq data) and gene expression (log_2_ FC of RNA-seq data) in T-ALL and thymus samples. Genes with promoters displaying increased H3K18la signals (log_2_ FC > 1 of ChIP-seq data, FDR < 0.05) and upregulated expression (log_2_ FC > 1 of RNA-seq data, FDR < 0.05) are marked as “Up”. Conversely, genes with promoters displaying decreased H3K18la signals (log_2_ FC < −1 of ChIP-seq data, FDR < 0.05) and downregulated expression (log_2_ FC < −1 of RNA-seq data, FDR < 0.05) are marked as “Down”. Genes recognized as T-ALL-related oncogenes in the COSMIC database or previous T-ALL research [16] are labeled as “Cancer-related”. The statistical significance of the correlation was calculated with Pearson correlation analysis. **D**. and **E**. The IGV genome browser displays H3K18la modification signals (D) and SLRs (E) on representative T-ALL-associated genes. **F**. Box plot shows transcriptional differences for genes listed in (E) between thymus and T-ALL samples, with statistical differences calculated using RNA-seq data. Each dot represents one sample. The outlier in the *NKX2-1* box plot represents the only sample of NKX2-1 subtype (P4) analyzed in this study. FDR values are shown in the plot. **G**. Gene set enrichment analysis of genes associated with SLRs in T-ALL (present in five or more T-ALL samples) and thymus samples (present in two or more thymus samples) for KEGG pathways. The top 5 most significantly enriched pathways in T-ALL or thymus with FDR < 0.1 are shown. **H**. Transcriptional differences between T-ALL and thymus (log_2_ FC). The genes were grouped into three classes, including thymus-specific SLR-associated genes (Thymus.SLRs), SLR-associated genes shared by thymus and T-ALL (Common.SLRs), and T-ALL-specific SLR-associated genes (T-ALL.SLRs). FC, fold change; KEGG, Kyoto Encyclopedia of Genes and Genomes; IGV, Integrative Genomics Viewer; NS, not significant.

Acknowledging the potential influence of H3K18la modifications in gene transcription modulation, we conducted a detailed analysis to uncover the differences between H3K18la modifications in T-ALL and normal thymus tissues, elucidating their connection with the transcriptional landscape of T-ALL. Our investigation revealed differential H3K18la patterns, with 24,161 sites exhibiting increased lactylation in T-ALL compared to normal T cells, while 7756 sites were downregulated (Table S4). Elevated H3K18la signals were predominantly localized to promoter regions, whereas reduced H3K18la levels were more commonly observed at intergenic regions (**Figure 4**A).

Comparative analysis highlighted a significant enrichment of H3K18la signals within genes associated with cell cycle pathways in T-ALL, contrasting with diminished signals in pathways governing T-cell immunity (Figure 4B). This suggests that H3K18la signals in T-ALL cells are primarily focused on regulating biological activities crucial to tumorigenesis while losing regulatory influences for normal T-cell functions. Correlations with transcriptional activity further revealed that many genes with increased H3K18la levels at T-ALL promoters exhibited upregulated transcription, implicating oncogenes such as *TAL1*, *LMO1*, *FLT3*, *WT1*, and *FAT1* (Figure 4C and D; Table S5).

Notably, T-ALL samples prominently exhibited elevated levels of H3K18la modifications forming distinctive SLRs which were particularly concentrated in the essential transcription factors (Figure S8). Among these pivotal factors highlighted in Figure 4E were *LMO2*, *CDK6*, *NKX2-1*, and *MLLT1*. Correspondingly, T-ALL samples displayed markedly heightened transcriptional activity of these genes (Figure 4F). Conversely, tumor suppressor genes such as *PTPRC* conspicuously lacked SLR signals in T-ALL (Figure 4E). Examination of SLR-associated genes in T-ALL unveiled the retention of thymic characteristics by tumor cells, evidenced by enrichment in GO categories associated with T/lymphocyte differentiation, in line with their thymic origin (Figure S9A). Moreover, a discernible shift in SLRs within T-ALL was noted from functions primarily associated with differentiation and immunological roles toward those characterized by tumor-associated transcriptional dysregulation and the regulation of small G protein receptor pathways (Figure 4G).

Significantly, genes exhibiting acquired or lost SLR signals in T-ALL demonstrated corresponding upregulated or downregulated transcription, respectively (with respective *P* values of 8E−06 and 1.4E−09, Wilcoxon signed-rank test) (Figure 4H), corroborating the regulatory influence of SLRs on transcription. Remarkably, the distinct SLRs identified in T-ALL exhibited high recurrence rates. A total of 48 SLRs were recurrently present in at least ten samples, encompassing over 76.92% of T-ALL cases analyzed in this study. Notably, these recurrent SLRs were associated with genes known to be implicated in cancer, as evidenced by the Catalogue of Somatic Mutations in Cancer (COSMIC), including *RALGDS*, *BCR*, *SH2B3*, *LMNA*, and *GATA2* (Figure S9B; Table S6). This underscores the important role of H3K18la in the transcriptional regulation of highly expressed genes in T-ALL, particularly those genes pivotal to the pathology of T-ALL.

### H3K18la positively associated with T-ALL proliferation through transcriptional regulation

**Figure 5:**
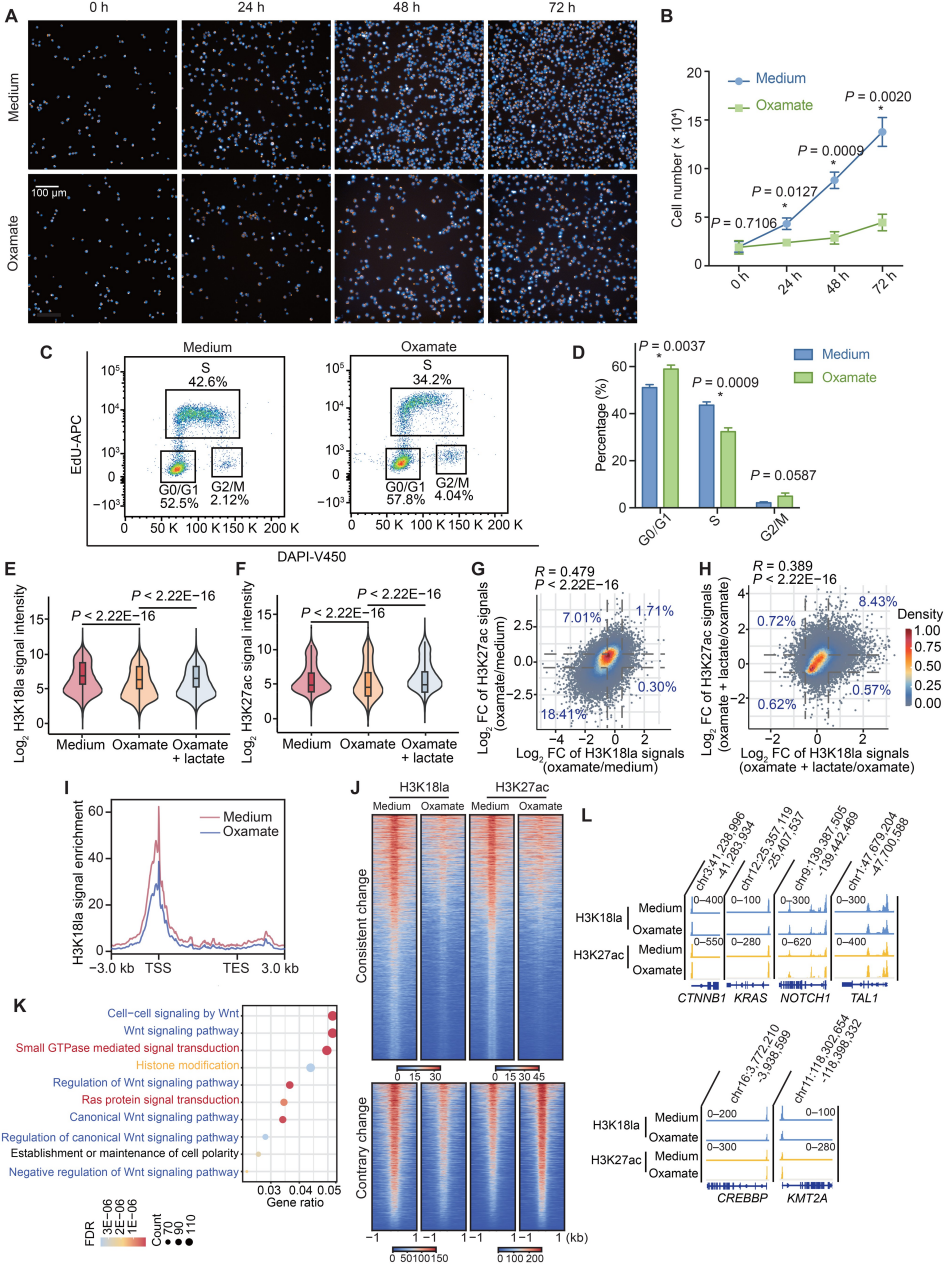
Disruption of H3K18la inhibited cell proliferation and interplayed with H3K27ac changes in transcriptional regulation **A**. Proliferation of Jurkat cells cultured in normal medium and medium supplemented with oxamate, visualized using high-content imaging. Nuclei were stained with Hoechst 33342 (blue fluorescence), and mitochondria were stained with TMRM (red-orange fluorescence). Scale bar, 100 µm. **B**. The proliferation results in (A) are depicted in a line graph, with statistical analysis performed using an independent *t*-test (*, *P* < 0.05). **C**. Cell cycle analysis of Jurkat cells treated with standard medium and medium supplemented with oxamate for 48 h. Proportions of cells in each phase of the cell cycle (G0/G1, S, and G2/M) were determined by flow cytometry. **D**. Bar plot shows the quantitative analysis results. Data are presented as mean ± SD (*n* = 3). Statistical significance was determined by the independent *t*-test (*, *P* < 0.05). **E**. and **F**. Violin plots display the signal intensities of H3K18la (E) and H3K27ac (F) in control group (normal medium), oxamate-treated group (medium supplemented with oxamate), and rescued group (medium supplemented with a combination of oxamate and lactate). **G**. and **H**. Scatter plots describe the correlation between changes in H3K18la ChIP-seq signals (log_2_ FC) and H3K27ac signals (log_2_ FC) after oxamate treatment (G) or oxamate + lactate treatment (H). Regions with both H3K18la and H3K27ac modifications are included in these analyses. Dashed lines indicate log_2_ FC of 0.5/−0.5. Pearson correlation coefficients (*R*) and *P* values, together with the percentage of genomic regions in each area of the plot, are shown. **I**. Distribution patterns of H3K18la signals in genes with decreased H3K18la intensity (log_10_ likelihood ratio > 3) and downregulated transcription (log_2_ FC < −1 RNA-seq data) upon oxamate treatment. **J**. Heatmaps exhibit the changes in H3K27ac signals in the regions with decreased H3K18la modifications upon oxamate treatment. **K**. Gene set enrichment analysis displays the enriched GO terms associated with genes with decreased H3K18la signals and increased H3K27ac signals following oxamate treatment. The top 10 most significantly enriched terms are presented. GO terms associated with the Wnt signaling pathway are labeled with blue, those associated with the RAS/GTPase signaling pathways are labeled in red, and those associated with epigenetic regulation are labeled in yellow. **L**. IGV browser displays exemplified gene regions with reduced H3K18la signals and elevated H3K27ac signals following oxamate treatment. TMRM, tetramethylrhodamine methyl ester.

To elucidate the impact of H3K18la on cell proliferation and gene expression, we employed the LDH inhibitor sodium oxamate to inhibit lactate production, leading to a reduction in H3K18la levels in cell lines. Subsequently, we restored H3K18la levels by reintroducing lactate. Results showed that oxamate treatment significantly inhibited tumor cell proliferation (**Figure 5**A and B). Flow cytometry analysis unveiled that cells were blocked in G0/G1 phase of cell cycle after the inhibition of lactate production (Figure 5C and D). Accordingly, oxamate treatment resulted in a significant decrease in H3K18la levels within the cell, with 20,376 regions showing reduced modifications (Table S7). Notably, these regions were predominantly enriched at promoters exhibiting high H3K18la levels before treatment (Figure S10A and B). We further confirmed the influence of lactate on cell proliferation using glycolysis inhibitor 2-deoxy-D-glucose (2-DG). Similarly, we observed significant suppression of cell proliferation after 2-DG treatment (Figure S11A and B), and cells were blocked at G0/G1 phase of cell cycle as revealed by flow cytometry (Figure S11C and D). Meanwhile, ChIP-seq analysis showed a significant genome-wide decrease in H3K18la modification after 2-DG treatment, including at the promoter regions of key genes in T-ALL (Figure S11E and F). The consistent findings observed with oxamate and 2-DG treatments supported that lactate could significantly influence cell proliferation and genome-wide H3K18la modifications. Notably, we found that reduction in H3K18la modification was accompanied by a significant decrease in H3K27ac levels, and both modifications were subsequently restored upon lactate repletion (*P* < 2.2E−16 for each test, Wilcoxon signed-rank test) (Figure 5E and F). Correlation analysis revealed a significantly positive correlation between H3K18la and H3K27ac modifications during the reduction and restoration processes (*P* < 2.2E−16 for each test, Pearson correlation test) (Figure 5G and H). Interestingly, we observed that H3K4me1 levels were also significantly decreased following oxamate treatment, but the changes were not significantly correlated with H3K18la (Figure S12A and B).

At the transcriptional level, we observed that out of 231 protein-coding genes displaying reduced transcription following oxamate treatment, 136 exhibited a significant decrease in H3K18la at their promoters, while 7 other genes showed reducded H3K18la within their gene bodies (Figure 5I; Table S8). Interestingly, while the changes in H3K27ac modification upon oxamate treatment coincided with those in H3K18la, conflicting changes were also noted. As depicted in Figure 5J, increased H3K27ac levels were observed at a total of 4120 sites where H3K18la levels decreased. This was particularly evident at promoters exhibiting high modification intensity for both H3K18la and H3K27ac before treatment (Figure S12C and D). Analysis revealed that genes within these regions were predominantly associated with pathways crucial for T-ALL, including the Wnt signaling transduction pathway, the RAS/GTPase signaling pathway, and epigenetic-related pathways (Figure 5K). Core genes within these pathways, such as *CTNNB1*, *KRAS*, *CREBBP*, and *KMT2A*, displayed a significant reduction in H3K18la and active compensation of H3K27ac at promoters following inhibitor treatment. Similarly, this phenomenon was observed in key transcription factor genes for T-ALL, including *TAL1* and *NOTCH1* (Figure 5L), with no substantial change at the transcriptional levels of these genes (Figure S12E). This suggests the existence of an interplay network involving mutual histone modifications in gene transcriptional regulation to maintain the transcription homeostasis in T-ALL.

As the H3K18la modification did not completely overlap with other histone modifications like H3K27ac or H3K4me1 as described above, we further examined whether H3K18la was associated with insulated neighborhood boundaries in the genome. By analyzing ChIP-seq data, we identified genomic regions colocalized by CTCF, SMC1, SMC3, and RAD21 as the insulated neighborhood boundaries. We observed that the insulated neighborhood boundaries were colocalized with H3K18la, in both DND41 and Jurkat cells (Figure S13). Further analysis showed that the insulated neighborhood boundaries were more intensively colocalized with H3K18la compared to H3K27ac. These results indicated that H3K18la might be involved in the maintenance of three-dimensional genome architecture to regulate gene transcription, which was independent of H3K27ac. However, a detailed analysis with experiments such as high-throughput chromosome conformation capture (Hi-C) would be needed to further investigate this mechanism.

### Gene transcription regulated by H3K18la was associated with prognosis in T-ALL patients

**Figure 6:**
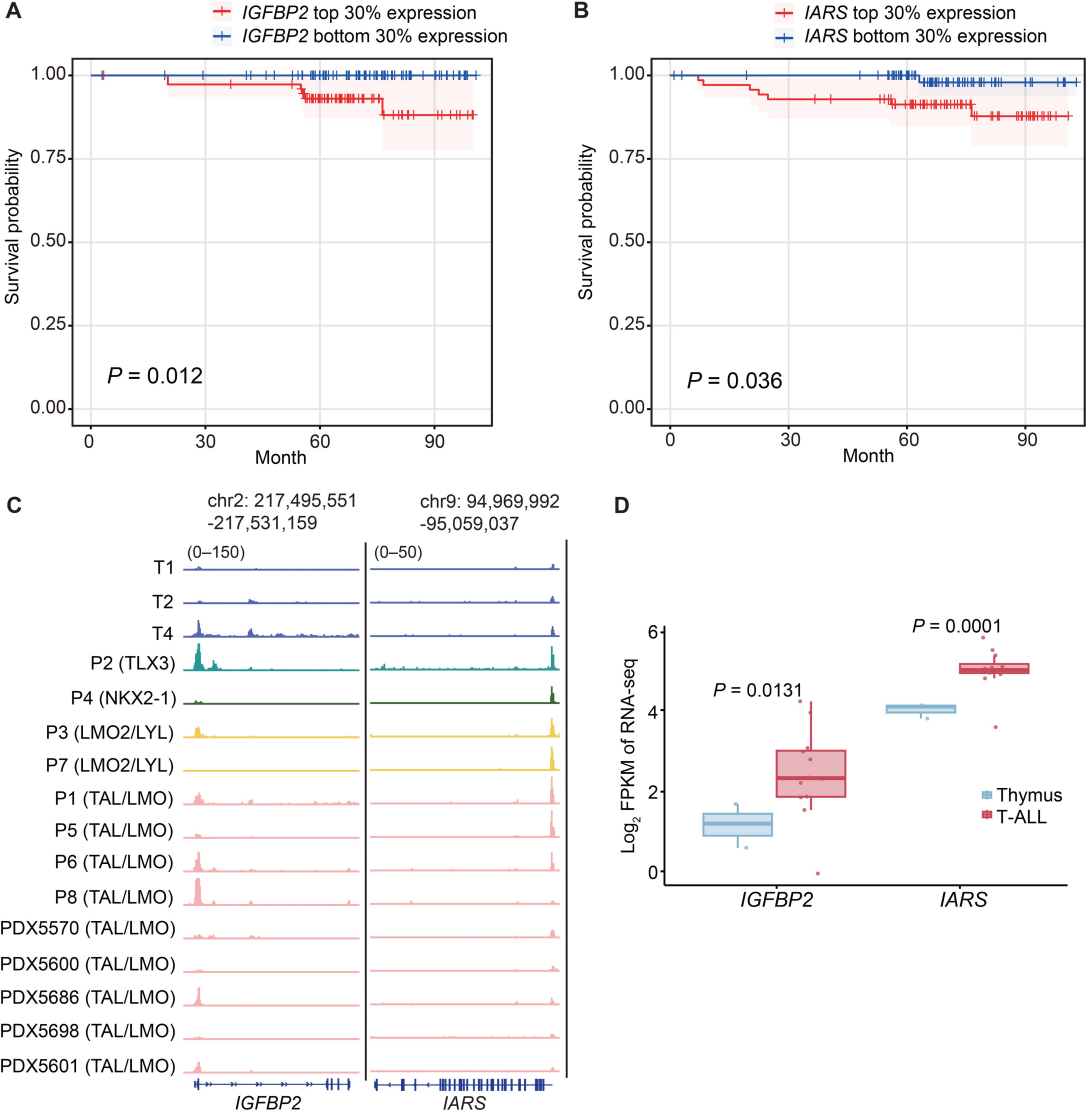
H3K18la-regulated gene transcription was associated with T-ALL prognosis **A**. and **B**. Overall survival rates for T-ALL patients grouped by transcription levels of H3K18la target genes *IGFBP2* (A) and *IARS* (B). Patients were categorized into high- and low-expression groups based on the top 30% (*IGFBP2*, *n* = 75; *IARS*, *n* = 70) and bottom 30% (*IGFBP2, n* = 74; *IARS*, n = 71) of gene expression levels, respectively. Statistical difference was determined by the log-rank test. **C**. Genome browser visualization of H3K18la profiles of target genes in normal T cells compared to T-ALL samples. **D**. Violin plot depicts the differences in gene transcription of *IGFBP2* and *IARS* between normal T cells and T-ALL samples. Statistical significance was determined by *t*-test.

We next investigated the potential role of H3K18la in patient prognosis. In this analysis, we focused on the genes transcriptionally regulated by H3K18la, defined as those showing both reduced H3K18la levels and decreased transcription upon oxamate treatment. Survival analysis identified two genes, *IGFBP2* and *IARS*, whose high transcriptional levels were significantly associated with inferior overall survival of T-ALL patients (*P* = 0.012 for *IGFBP2* and 0.036 for *IARS*, log-rank test) (**Figure 6**A and B). Further analysis of ChIP-seq and RNA-seq data revealed increased H3K18la levels at the promoters of these two genes in T-ALL patients compared to normal T cells (Figure 6C). This increase was coupled with higher transcription (Figure 6D), thus supporting the role of H3K18la modification in the transcriptional activation of these genes. These findings suggest that elevated H3K18la levels are associated with inferior prognosis in T-ALL through transcriptional regulation.

## Discussion

The Warburg effect stands as one of the most fundamental metabolic reconfigurations in all types of cancer [3]. With the heightened glycolysis rate within cells, lactate production increases. Only recently have researchers uncovered histone lactylation and begun to connect accumulated lactate with epigenetic regulation within cells [4], offering a fresh perspective on this century-old metabolic rewiring. However, the implications of histone lactylation modifications in the cancer genome remain largely unexplored. In this study, we conducted a genome-wide analysis of H3K18la in pediatric T-ALL. Given that dysregulation of epigenetic modifications and transcriptional regulation is a core driver aberration in this malignancy [16,17], T-ALL serves as an ideal model for investigating the role of histone lactylation in transcriptional regulation through metabolic reconfiguration-induced epigenetic changes.

In this study, we showed that H3K18la modification was increased in tumor cells and associated with cell proliferation, establishing its role in T-ALL leukemogenesis. We observed notable differences in global H3K18la patterns between T-ALL samples and normal T cells obtained from thymus tissues, suggesting that metabolic shifts trigger widespread epigenetic alterations. Our results reveal that H3K18la modifications in T-ALL are significantly enriched at gene promoters, including many oncogenic transcription factor genes and key tumorigenic genes, and are closely associated with elevated gene expression. Considering the pivotal role of transcriptional dysregulation in T-ALL pathogenesis, we proposed that lactylation modifications may play a significant role in T-ALL through transcriptional regulation. In line with this, our analysis further identified two potential targets of H3K18la modification, *IGFBP2* and *IARS*, whose high transcriptional levels were significantly associated with poor prognosis in T-ALL. Notably, *IGFBP2* is an established oncogene in various tumors including myeloid leukemia, gliomas, breast, and prostate cancer, and orchestrates multiple cancer signaling pathways such as PI3K-AKT, NF-κB, and EGFR/STAT3 [31–33]. On the other hand, the role of *IARS* remains less defined in tumors. These findings offer novel insights into tumor pathogenesis by highlighting a regulatory axis encompassing metabolic reprogramming, epigenetic remodeling, and transcriptional reconfiguration.

By integrating other histone modifications with well-established roles in transcriptional regulation, we demonstrated that H3K18la correlates with active transcriptional regions in both T-ALL samples and normal T cells, including promoters and some enhancers, consistent with previous reports [12]. Notably, H3K18la shows a strong correlation with promoter activity, present at nearly all active promoters and positively associated with Pol II occupancy. The increased H3K18la signals at gene promoters correlate not only with enhanced Pol II binding at transcription start sites (TSSs) but also with extended gene body signals, providing further evidence that H3K18la enrichment at TSSs may facilitate the release of paused Pol II and thereby enhance transcriptional elongation.

Regarding enhancer regions, our findings align with previous studies indicating a significant overlap between H3K18la and classical enhancer markers such as H3K27ac or H3K4me1, along with a positive correlation with chromatin accessibility [12,13]. Nevertheless, the role of H3K18la in distal genomic regions requires further characterization. Intriguingly, our study suggests that H3K18la may serve as regulatory clusters (SLRs) exhibiting SE characteristics in the genome. We demonstrated that SLRs are a common phenomenon across various tissues, regulating genes crucial to cell identity and essential functions. Consequently, we observed a shift in SLRs within the genome between normal T cells and T-ALL samples, associated with a transition in cellular function from driving differentiation and immune responses to promoting transcriptional dysregulation, thus contributing to leukemogenesis.

Another intriguing finding pertains to the dynamic interplay between H3K18la and other histone modifications. Previous research has suggested that lactylation and acetylation may involve shared writers and erasers [4,34,35]. We demonstrated that H3K18la and H3K27ac exhibit similar patterns of genomic distribution, with their modification levels positively correlated. Our *in vitro* experiments revealed that manipulation of H3K18la levels resulted in corresponding changes in H3K27ac, indicating overlapping regulatory mechanisms governing these two modifications, allowing for their simultaneous marking and removal. Surprisingly, we observed increased H3K27ac modifications in a subset of the genome when H3K18la decreased, suggesting the presence of distinct writers/erasers for these modifications that do not overlap. Notably, these regions included several critical genes in T-ALL, such as *TAL1* and *NOTCH1*. This observation underscores the intricate epigenetic regulatory network within cells, orchestrating multiple histone modifications to maintain transcriptional homeostasis.

With advancements in mass spectrometry techniques, an increasing number of novel histone modifications have been identified [36]. These emerging histone marks are likely to extend beyond their individual characteristics. It is essential to understand how these marks contribute to the existing complex regulatory networks. Taking H3K18la analyzed in the current study as an example, we propose that newly identified modifications may not operate in isolation. Their functions are intertwined with those of established core histone modifications. Since therapies targeting epigenetic modifications represent one of the major focuses of current research, strategies aimed at targeting multiple epigenetic modifications may offer enhanced benefits for patients.

In summary, we demonstrated how metabolic reprogramming can profoundly impact epigenetic remodeling and participate in the transcriptional regulation of key driver genes in T-ALL through H3K18la, thus involved in leukemogenesis. Detailed analysis of H3K18la’s association with clinical characteristics of T-ALL, including early T-cell precursor (ETP) status and molecular subtypes, would further clarify its role in this malignancy. Meanwhile, it is noteworthy that other histone lactylation modifications besides H3K18la might also play important roles in bridging the metabolism and epigenetic regulation, like H3K9la and H3K14la, which remain to be further investigated. Our research focuses on the interplay between H3K27ac and H3K18la as a starting point, highlighting that tumor transcriptional regulation is an integrated system involving complex interactions among histone modifications. Developing and applying robust single-cell ChIP-seq methods to simultaneously analyze multiple histone modifications (including H3K18la and others) would provide key information to understand the combinatorial functions of these histone modifications. Furthermore, while our primary focus is on tumor cells, it is important to consider the broader implications of the “lactate shuttle” mechanism [37], which may play a significant role in the tumor environment and warrants further investigation. With these insights, we believe that our findings expand the understanding of the interplay between tumor metabolism and epigenetic remodeling in T-ALL and lay a theoretical foundation for the development of epigenetic therapeutic strategies in this malignancy.

## Materials and methods

### Patient samples

Bone marrow samples were collected from patients diagnosed with T-ALL at the Shanghai Children’s Medical Center (SCMC) during 2011–2013. The age distribution of these patients included three patients aged 3–10 years and five aged 10–16 years. Flow cytometry revealed that tumor cells accounted for > 90% of the cellular composition for all samples analyzed. Thymic samples were obtained from congenital heart disease surgeries conducted at the SCMC. For molecular subtyping of the T-ALL samples, RNA-seq data were analyzed using a gene expression pattern-based subtype classification, developed in-house with a recurrent neural network (RNN) model (Cui et al, unpublished data).

### PDX generation

One to two million primary T-ALL tumor cells were intravenously injected into M-NSG mice 24 h after irradiation. The tumor burden was evaluated by quantifying the percentage of human CD45^+^CD7^+^ cells in the peripheral blood via flow cytometry. Subsequently, xenografted cells were harvested from the spleens of mice that developed leukemia, and samples with a tumor cell proportion greater than 90% were utilized for downstream analyses.

### Cell culture

The Jurkat and DND41 cell lines were kindly provided by Prof. Hudan Liu (Frontiers Science Center for Immunity and Metabolism, Medical Research Institute of Wuhan University, China) and were cultured in an incubator at 37°C with 5% CO_2_. Growth was supported by Roswell Park Memorial Institute (RPMI) 1640 medium (Catalog No. C11875500BT, Gibco, Grand Island, NY) supplemented with 10% fetal bovine serum (FBS; Catalog No. 10099141C, Gibco). Cells were routinely tested for potential mycoplasma contamination. For the sodium oxamate inhibition experiment, Jurkat cells were cultured for 48 h in standard medium, medium containing 50 mM sodium oxamate (Catalog No. S6871, Selleck, Houston, TX), and medium supplemented with both 50 mM sodium oxamate and 10 mM L-sodium lactate (Catalog No. 71718, Sigma, St. Louis, MO). In the 2-DG inhibition experiment, Jurkat cells were cultured for 24 h in normal medium and medium supplemented with 5 mM 2-DG (Catalog No. S4701, Selleck). Following incubation, cells were harvested for subsequent sequencing experiments.

### Lactate assay

One million cells were collected and lysed using 1 ml methanol that had been pre-cooled to −80°C. Cells were incubated for 30 min at 4°C to ensure thorough lysis and then centrifuged at 15,000 r/min for 30 min at 4°C. The supernatant was collected for LC-MS/MS analysis. Each sample included three biological replicates, and outliers were excluded during data processing.

### Cell proliferation assays

Jurkat cells with equal initial cell numbers were cultured in standard medium and medium supplemented with 50 mM sodium oxamate. Cell proliferation was assessed at 0 h, 24 h, 48 h, and 72 h. To identify live cells, nuclei were stained with Hoechst 33342 (Catalog No. 62249, Thermo Fisher Scientific, Waltham, MA) with excitation at 350 nm, and mitochondria were stained with tetramethylrhodamine methyl ester (TMRM; Catalog No. I34361, Thermo Fisher Scientific) with excitation at 574 nm, according to the manufacturer’s instructions. High-content imaging was performed using the PerkinElmer Operetta CLS high-content analysis system. Images were captured in multiple fields of view to ensure a representative sample of the cell population. Live cells were quantified based on the presence of both Hoechst 33342-stained nuclei and TMRM-stained mitochondria, with cell counting performed using automated image analysis software.

### Cell cycle analysis

Cells were initially cultured in standard medium and medium containing 50 mM sodium oxamate for 48 h. Subsequently, the medium was replaced with fresh medium containing 5-ethynyl-2′-deoxyuridine (EdU) at a final concentration of 10 µM, and the cells were incubated for an additional 2 h. EdU incorporation was detected using the Click-iT EdU Alexa Fluor 647 Flow Cytometry Assay Kit (Catalog No. C10424, Thermo Fisher Scientific) in accordance with the manufacturer’s protocol. Then, the samples were stained with 4′,6-diamidino-2-phenylindole (DAPI) and analyzed by flow cytometry.

### ChIP-seq experiment

Native ChIP-seq was performed following standard protocols [38]. Cell pellets (1 × 10^6^ to 1 × 10^7^ cells) were resuspended in 50 μl nuclear isolation buffer (Catalog No. NUC-10, Sigma) containing protease inhibitors. Chromatin digestion was performed using 4–5 U MNase (Catalog No. M0247, New England Biolabs, Ipswish, MA) for 9–20 min, and the reaction was stopped with 100 μM ethylenediaminetetraacetic acid (EDTA). The nuclear membranes were lysed using 1% Triton X-100 and 1% deoxycholate. DNA fragments, predominantly mononucleosome-sized as confirmed by agarose gel electrophoresis, were subjected to immunoprecipitation using a buffer containing 20 mM Tris-HCl (pH 8.0), 2 mM EDTA, 150 mM NaCl, 0.1% Triton X-100, protease inhibitors, and 1 mM phenylmethylsulfonyl fluoride (PMSF; Catalog No. P7626, Sigma). Chromatin was pretreated with an equal mix of Protein A (Catalog No. 10002D, Life Technologies, Carlsbad, CA) and G Dynabeads (Catalog No. 10004D, Life Technologies). Concurrently, anti-H3K18la (Catalog No. PTM-RM1427, PTM Bio, Hangzhou, China) or anti-H3K27ac (Catalog No. ab4729, Abcam, Cambridge, UK) antibodies were conjugated with another mixture of Protein A and G Dynabeads for 4 h. This preparation was then incubated overnight at 4°C. The beads were washed with two cycles of low-salt buffer wash [20 mM Tris-HCl pH 8.0, 2 mM EDTA, 150 mM NaCl, 1% Triton X-100, and 0.1% sodium dodecyl sulfate (SDS)] followed by two cycles of high-salt buffer wash (20 mM Tris-HCl, pH 8.0, 2 mM EDTA, 500 mM NaCl, 1% Triton X-100, 0.1% SDS). DNA was eluted using 100 mM NaHCO_3_ and 1% SDS at 65°C for 2 h, treated with RNase A (Catalog No. B500474, Sangon Biotech, Shanghai, China) at 37°C for 30 min, purified with phenol-chloroform-isoamyl alcohol, ethanol-precipitated, and finally dissolved in elution buffer (EB; Catalog No. 19086, Qiagen, Hilden, Germany). The purified DNA was used for sequencing library preparation followed by high-throughput sequencing. *Drosophila* DNA was used as a spike-in control (Catalog Nos. 61686 and 53086, Active Motif, Carlsbad, CA) except for the H3K27ac profiling for PDX5570.

### RNA-seq experiment

Total RNA was extracted from cells using TRIzol reagent (Catalog No. 15596026, Invitrogen, Carlsbad, CA). For the sodium oxamate inhibition experiment, total RNA was extracted from an equal number of cells, followed by addition of a diluted External RNA Controls Consortium (ERCC) RNA spike-in mix (Catalog No. 4456740, Invitrogen). RNA integrity was assessed using the Agilent 2100 Bioanalyzer system, and only samples with RNA integrity number (RIN) > 6 were used for library construction. Library preparation was performed using two distinct approaches. For patient and thymus samples, Ribo-Zero strand-specific libraries were prepared following ribosomal RNA (rRNA) depletion from total RNA using the NEBNext rRNA Depletion Kit (Catalog No. E6310, New England Biolabs). For cell lines from the sodium oxamate inhibition experiment, mRNA sequencing (mRNA-seq) libraries were constructed after poly(A) mRNA isolation from total RNA using the NEBNext Poly(A) mRNA Magnetic Isolation Module (Catalog No. E7490, New England Biolabs). All libraries were generated in accordance with the manufacturer’s recommendations using the NEBNext Ultra RNA Library Prep Kit for Illumina (Catalog No. E7530, New England Biolabs) and subsequently sequenced on the Illumina NovaSeq 6000 system.

### ChIP-seq data processing

Following the removal of low-quality reads and adapters, quality control was executed using FastQ Screen (v0.13.0) [39] and FastQC (v0.11.9). For PDX samples, mouse genomic sequences were separated using BBSplit (v38.90). Reads were then aligned to the human reference genome (hg19) using the Burrows–Wheeler Aligner (BWA; v0.7.17-r1188) [40], with non-uniquely mapped reads discarded. Duplicate reads were marked with the MarkDuplicates feature in Picard (v2.22.9). Peak calling was performed with MACS2 (v2.2.6) [41], applying the parameters “-f BAMPE -g hs -B”. Peaks overlapping with regions on the ENCODE blacklist were excluded from the downstream analysis. Annotation of identified ChIP-seq peaks was conducted within 100 kb of the nearest gene, employing the ChIPseeker software (v1.36.0) [42] and the gencode.v19.annotation. To visualize the normalized read pile-up within the Integrative Genomics Viewer (IGV; v2.16.2) [43], bigWig files standardized by spike-in were generated using the bamCoverage tool of deepTools (v3.4.3) [44].

### Differential analysis of Chip-seq data

Before comparing H3K18la signal intensities at peak regions across different samples, downsampling was performed to account for the effect of sequencing depth on peak detection. BEDTools (v2.26.0) [45] multicov was utilized to calculate the read counts for H3K18la at each peak based on the sample sequencing depth. Subsequently, peak normalization was conducted using spike-in controls. Differential analysis of H3K18la signals between thymic and T-ALL cells was performed using the R package DiffBind (v3.10.1, http://bioconductor.org/packages/release/bioc/vignettes/DiffBind/inst/doc/DiffBind.pdf) [46]. Within *dba.count()*, the “summit” parameter was set to 400 to standardize consensus peak widths to 801 bp and to compute sequencing read counts for samples at these peaks. The *dba.normalize()* function was then applied for normalization of ChIP-seq read counts with the setting “normalize = DBA_NORM_LIB, spikein = TRUE”, leveraging *Drosophila* spike-in reads for standardization. Sample comparisons were established using the *dba.contrast()* function, and differential accessibility was statistically assessed via the DBA_DESEQ2 method. Peaks with false discovery rate (FDR) < 0.05 were considered statistically significant. Visualization of the normalized data was performed using *dba.plotHeatmap()* for unsupervised clustering heatmaps and *dba.plotPCA()* for PCA plots. In the sodium oxamate inhibition experiment, differential peak analysis was conducted using bedGraph files generated by MACS2 software for H3K27ac and H3K18la, along with their corresponding input samples. The MACS2 *bdgdiff* function was employed with the parameters “-d1” and “-d2” to normalize the target library sizes based on the *Drosophila* spike-in library. Peaks with log_10_ likelihood ratio > 3 were identified as differentially enriched.

### Heatmaps and density plots of ChIP-seq data

In DND41 cells, deepTools (v3.4.3) was used to compare ChIP-seq and ATAC-seq signal intensities across promoter and enhancer regions. This analysis was further extended to include comparisons of H3K18la and H3K27ac signals in T-ALL patient samples and during lactate inhibition induced by sodium oxamate. The *computeMatrix* and *plotHeatmap* functions were employed to compute and visualize signal enrichments within targeted regions, utilizing bigWig formatted data. For the comparison of DND41 and Jurkat signals at insulated neighborhood boundaries, the ChIP-seq signals for CTCF, SMC1, SMC3, and RAD21 were computed using the *computeMatrix* function. These signals were then clustered using the *k*-means clustering algorithm, which partitions the data into distinct clusters based on the similarity of signal profiles. Regions where CTCF, SMC1, SMC3, and RAD21 co-localized were identified as insulated neighborhood boundaries. Subsequently, the same approach was applied to compute and visualize the enrichment of H3K18la and H3K27ac modifications within these regions. The SMC1 data for DND41 (GEO: GSM5282081) and the CTCF data for Jurkat (GEO: GSM1689152) were retrieved from the Gene Expression Omnibus. Data for CTCF, SMC3, and RAD21 in DND41, as well as for SMC3 and RAD21 in Jurkat, were sourced from the ENCODE project database (https://www.encodeproject.org) [25].

### Chromatin state analysis using ChromHMM

The ChromHMM software (v1.19) [47] was employed to identify H3K18la-associated chromatin states in DND41 cells. ChIP-seq data for histone modifications, including H3K4me3, H3K4me1, H3K27ac, H3K36me3, H3K27me3, and H3K9me3, were obtained from the ENCODE project database (https://www.encodeproject.org) [25]. The peak positions of classic histone modifications and H3K18la in DND41 were extracted from the BED files and binarized at a 1000-bp resolution using the BinarizeBed command. Models with varying chromatin state numbers (ranging from 10 to 18) were learned from the binarized data via the *LearnModel* function. The ChromHMM state labels were assigned based on the combinatorial patterns of the specified histone marks.

### Identification of SLRs and SEs

The ROSE software (v0.1) [30,48] was employed to identify SLRs and SEs by merging peaks within 12.5 kb of H3K18la or H3K27ac. Signals within promoter regions were excluded from this analysis. Genes associated with SLRs shared by ≥ 5 T-ALL patients were considered as tumor-associated SLR genes. Conversely, genes associated with SLRs detected in ≥ 2 thymus samples were classified as thymus-associated SLR genes. For SLR analysis, data for muscle samples were collected from the Gene Expression Omnibus: Cleavage Under Targets and Tagmentation (CUT&Tag) data (GEO: GSE195854) [12], RNA-seq data (GEO: GSE144133) [49], and macrophage ChIP-seq and RNA-seq data (GEO: GSE115354) [4].

### RNA-seq data processing

Quality control of the raw sequencing data was performed using FastQ Screen (v0.13.0) and FastQC (v0.11.9). For PDX samples, mouse genomic sequences were removed using the BBSplit tool (v38.90). Then, reads were aligned to either the human genome (hg19) or hybrid human (hg19)–ERCC genome using Spliced Transcripts Alignment to a Reference (STAR) software (v2.7.1a) [50]. Read counts within each gene were computed using HTSeq (v0.11.2) [51]. The quantification of gene transcription was conducted by calculating fragments per kilobase of transcript per million mapped reads (FPKM). The data from the sodium oxamate inhibition experiment were normalized through the RUVSeq R package (v1.34.0) [52], utilizing the ERCC synthetic RNA controls.

For differential expression analysis of RNA-seq data between thymic cells and T-ALL cells, DESeq2 (v1.40.2) [53] was used to assess statistical significance. Genes with |log_2_ fold change (FC)| > 1 and FDR < 0.05 were deemed significant. In the analysis of the sodium oxamate inhibition experiment, the *exactTest* function within edgeR (v3.42.4) was employed with significant genes defined as having |log_2_ FC| > 1.

### Functional enrichment analysis

Functional enrichment analysis was conducted utilizing the *enrichGO* and *enrichKEGG* functions within the clusterProfiler R package (v4.0.5) [54,55]. Differential regions associated with T-ALL *vs.* thymus, identified by DiffBind, were functionally annotated via the chipenrich R package (v3.11.0) [56]. FDR < 0.1 was considered statistically significant.

## Ethical statement

The study received approval from the Institutional Review Board of the Shanghai Children’s Medical Center, China (Approval No. SCMCIRB-Y2019034), and informed written consents were obtained for all participants.

## Supplementary Material

qzaf029_Supplementary_Data

## Data Availability

The RNA-seq and ChIP-seq data generated from T-ALL patients and thymus samples as well as from DND41 and Jurkat cell lines have been deposited in the Genome Sequence Archive for Human [57] at the National Genomics Data Center (NGDC), China National Center for Bioinformation (CNCB) (GSA-Human: HRA006791 for T-ALL patients and thymus samples; HRA012594 for DND41 and Jurkat cell lines), and are publicly accessible at https://ngdc.cncb.ac.cn/gsa-human. The RNA-seq and ChIP-seq data from the DND41 and Jurkat cell lines are aslo available in the Gene Expression Omnibus (GEO: GSE263697 for DND41 cell line; GSE263584 for Jurkat cell line).

## CRediT author statement

**Wenyan Wu:** Formal analysis, Investigation, Methodology, Visualization, Validation, Writing – original draft, Writing – review & editing. **Jingyi Zhang:** Resources, Investigation. **Huiying Sun:** Formal analysis, Methodology. **Xiaoyu Wu:** Investigation. **Han Wang:** Investigation, Methodology. **Bowen Cui:** Formal analysis, Visualization. **Shuang Zhao:** Formal analysis, Data curation. **Kefei Wu:** Resources. **Yanjun Pan:** Resources. **Rongrong Fan:** Formal analysis. **Ying Zhong:** Formal analysis, Data curation. **Xiang Wang:** Resources. **Ying Wang:** Formal analysis. **Xiaoxiao Chen:** Resources. **Jianan Rao:** Formal analysis. **Ronghua Wang:** Resources, Project administration. **Kai Luo:** Resources. **Xinrong Liu:** Resources. **Liang Zheng:** Investigation. **Shuhong Shen:** Resources, Funding acquisition. **Meng Yin:** Resources, Supervision, Funding acquisition. **Yangyang Xie:** Resources, Supervision. **Yu Liu:** Conceptualization, Supervision, Funding acquisition, Writing – review & editing. All authors have read and approved the final manuscript.

## ORCID

0009-0000-8097-3483 (Wenyan Wu)

0000-0002-9549-1337 (Jingyi Zhang)

0009-0001-3712-263X (Huiying Sun)

0009-0005-4325-6174 (Xiaoyu Wu)

0009-0005-1960-4474 (Han Wang)

0000-0003-4467-1669 (Bowen Cui)

0000-0002-2779-5590 (Shuang Zhao)

0000-0003-0599-1272 (Kefei Wu)

0009-0005-2862-7977 (Yanjun Pan)

0009-0007-7449-2406 (Rongrong Fan)

0009-0008-4402-1926 (Ying Zhong)

0009-0004-4105-8839 (Xiang Wang)

0009-0008-9546-8939 (Ying Wang)

0009-0004-9099-1588 (Xiaoxiao Chen)

0009-0006-0936-8196 (Jianan Rao)

0009-0003-5032-699X (Ronghua Wang)

0000-0002-4567-4039 (Kai Luo)

0009-0006-1279-6378 (Xinrong Liu)

0000-0002-5346-4111 (Liang Zheng)

0000-0002-0001-642X (Shuhong Shen)

0000-0002-4455-2005 (Meng Yin)

0009-0006-1606-3585 (Yangyang Xie)

0000-0001-8928-2558 (Yu Liu)
