# Supplementary material for: Glycolysis Induces Abnormal Transcription Through Histone Lactylation in T-cell Acute Lymphoblastic Leukemia
Source: Genomics Proteomics Bioinformatics. 2025 Apr 7;23(2):qzaf029. doi: 10.1093/gpbjnl/qzaf029 (PMC12402983; doi:10.1093/gpbjnl/qzaf029)
Supplement: qzaf029_Supplementary_Data [file qzaf029_supplementary_data.zip › supplementary material captions.docx]

**Supplementary material**

**Figure S1 Characteristics of histone H3K18la modification in normal T cells and pediatric T-ALL tumor cells**

**A.** and **B.** Comparison of the peak numbers (A) and signal coverage (B) of H3K18la ChIP-seq sequencing between T-ALL and thymus samples, with statistical differences assessed using the Wilcoxon signed-rank test. Data are down sampled before comparison. **C.** Distribution of H3K18la modification in gene regions in T-ALL and thymus cells.

**Figure S2 Genomic features of H3K18la modification compared to other histone modifications**

**A.**–**E.** The Venn diagrams elucidate intersections of genomic regions with modification of H3K18la and H3K4me3 (A), H3K4me1 (B), H3K27ac (C), H3K9me3 (D), and H3K27me3 (E). **F.** The left graph presents the percentage of the genome encompassed by H3K18la peaks that do not overlap with histone modifications of H3K4me3, H3K4me1, H3K27ac, H3K9me3, H3K27me3, and H3K36me3. The right bar chart shows the percentage of genomic regions with accessible chromatin in each group, as identified by ATAC-seq. A total of four groups are compared, including genomic regions with H3K18la modification alone, H3K18la together with H3K27ac, H3K18la with H3K4me1, and H3K18la with H3K4me3. **G.** Plots of signal intensity for modifications of H3K4me1, H3K27ac, H3K18la, and chromatin accessibility in enhancer regions defined by H3K4me1. The genome regions were separated into two groups based on the presence of H3K18la modification. **H.** Enhancer regions defined by H3K4me1 are categorized by varying levels of H3K18la modification signal intensity. The plots illustrate the signal intensity for modifications of H3K18la, H3K4me1, H3K27ac, and chromatin accessibility within these groups.

**Figure S3 Profiles of H3K18la and H3K27ac modifications at promoters in T-ALL tumor samples**

The figure displays the signal intensity distribution of H3K18la and H3K27ac modifications within promoter regions in T-ALL. Promoters are separated into three groups: with concurrent H3K18la and H3K27ac modifications, and with H3K18la or H3K27ac modifications alone.

**Figure S4 Profiles of H3K18la and H3K27ac modifications at enhancers in T-ALL tumor samples**

The figure shows signal intensity distributions for H3K18la and H3K27ac modifications within H3K27ac-defined enhancer regions in T-ALL. The enhancers are separated into two groups upon the presence of H3K18la modification in these regions.

**Figure S5 Transcriptional differences of genes with H3K18la and/or H3K27ac modification at promoters in T-ALL**

Genes are grouped upon the histone modification of promoters. H3K18la & H3K27ac means that the promoter regions are marked with both H3K18la and H3K27ac. H3K18la means that only H3K18la modification is observed. H3K27ac means that only H3K27ac is observed at promoters. Statistical analysis was conducted using the Wilcoxon signed-rank test. Only T-ALLs with RNA-seq data are showed.

**Figure S6 SLRs and SEs in the genome of normal and tumor cells**

**A.** In DND41 cells, signals for H3K27ac modification within a 12.5 kb range are merged and ranked, indicating SEs. Genes overlapping with T-ALL oncogenes listed in the COSMIC database, and those associated with T/lymphocyte differentiation according to the Gene Ontology database were labeled. **B.** Venn diagram depicts the intersection of genes related to SEs and SLRs in DND41 cells. **C.** Gene set enrichment analysis for the genes associated with both SLRs and SEs as indicated in (B). The top ten most significantly enriched pathways are shown. **D.** Gene set enrichment analysis illustrates the enrichment of genes associated with SLRs in thymic tissue, depicting the top ten pathways with the most significant FDR values. SEs, super-enhancers.

**Figure S7 Genes associated with SLRs in normal T cells**

In thymus tissue, H3K18la modification signals within a 12.5 kb region are ranked and labeled with genes associated with hematological malignancies from the COSMIC database. Genes that are specific to thymus and not associated with T-ALL’s SLRs are highlighted in green.

**Figure S8**  **Genes associated with SLRs in T-ALLs**

H3K18la modification signals within a 12.5 kb region are ranked and labeled with genes associated with hematological malignancies from the COSMIC database. Genes uniquely associated with SLRs in T-ALL but not normal T cells from thymus are highlighted in green.

**Figure S9 Genes associated with recurrent SLRs in T-ALL**

**A.** Gene set enrichment analysis displays Gene Ontology categories associated with T-ALL SLRs identified in at least five samples, listing the top ten pathways with the most significant FDR for Gene Ontology enrichment. **B.** Wiggle plots show examples of cancer related genes associated to highly recurrent (≥ 10) T-ALL SLRs.

**Figure S10 Alterations in H3K18la modification after oxamate treatment**

**A.** and **B.** The figures show the original modification intensities (A) and genomic distribution (B) of H3K18la and H3K27ac modifications in Jurkat cells before treated with oxamate. The regions are grouped based on the changes of H3K18la modification after oxamate treatment.

**Figure S11 Inhibition of glycolysis by 2-DG reduces cell proliferation via downregulation of H3K18la**

**A.** and **B.** Proliferation of Jurkat cells cultured in standard medium and medium supplemented with 2-DG, visualized by high-content imaging. Nuclei were stained with Hoechst 33342 (blue fluorescence) and mitochondria with TMRM (red-orange fluorescence). Scale bar = 100 µm (A). The bar plots display cell counts at different time points upon treatment. Data are presented as mean ± SD from three independent experiments, with statistical analysis conducted using an independent *t*-test (B). * means *P* < 0.05. **C** and **D.** Cell cycle analysis of Jurkat cells treated with normal medium and medium supplemented with 2-DG for 24 h. The distribution of cells across G0/G1, S, and G2/M phases was assessed by flow cytometry (C). Quantitative analysis is shown in a bar graph. Data are presented as mean ± SD from three independent experiments, with statistical significance determined by an independent *t*-test (D). **E.** Violin plot shows the difference in genome wide H3K18la modifications between normal medium and 2-DG treatment. **F.** Wiggle plots show the reduction of H3K18la signal in genes associated with T-ALL upon 2-DG treatment. 2-DG, 2-deoxy-D-glucose.

**Figure S12 Changes in H3K27ac and H3K4me1 modifications following oxamate treatment**

**A.** The violin plot illustrates the changes in H3K4me1 modification following oxamate treatment. Statistical significance was assessed using the Wilcoxon signed-rank test. **B.** The scatter plot depicts the correlation between changes in H3K18la ChIP-seq signals (log_2_FC) and H3K4me1 signals (log_2_FC) after oxamate treatment. **C.** and **D.** The plots illustrate the modification intensities (C) and genomic distribution (D) of H3K18la and H3K27ac modifications before oxamate treatment. Only regions with H3K27ac changes are show. Down regulation means decrease in both H3K18la and H3K27ac modifications after oxamate treatment. Up regulation means decreased H3K18la but increased H3K27ac modification after oxamate treatment. **E.** Bar graph displays transcriptions changes for genes associated with decreased H3K18la and increased H3K27ac modification following oxamate addition, as presented in Figure 5I. Dash lines in red indicate a log_2_FC of transcription greater than 1 or less than −1 upon treatment.

**Figure S13 Co-localization of H3K18la with insulated neighborhood boundaries**

**A.** and **B.** The figure shows the signal intensities of H3K18la and H3K27ac within insulated neighborhood boundary regions in DND41 (A) and Jurkat (B) cells. Insulated neighborhood boundary regions were defined by ChIP-seq data against CTCF, SMC1, SMC3, and RAD21 proteins.

**Table S1 Clinic and experiments information of T-ALLs analyzed in this study**

**Table S2 Quality control metrics for ChIP-seq and RNA-seq data**

**Table S3 Raw data from LC-MS analysis of lactate molecules**

**Table S4 Differential analysis of H3K18la modification in T-ALL versus thymus across consensus peaks**

**Table S5 Differential gene expression analysis between T-ALL and thymus using DESeq2**

**Table S6 Genes associated with SLRs in thymus and T-ALLs**

**Table S7 Genomic locations showing decreased H3K18la modification following oxamate addition**

**Table S8 Changes in gene transcription following the addition of oxamate**
