# Supplementary figures and images for: Glycolysis Induces Abnormal Transcription Through Histone Lactylation in T-cell Acute Lymphoblastic Leukemia
Source: Genomics Proteomics Bioinformatics. 2025 Apr 7;23(2):qzaf029. doi: 10.1093/gpbjnl/qzaf029 (PMC12402983; doi:10.1093/gpbjnl/qzaf029)

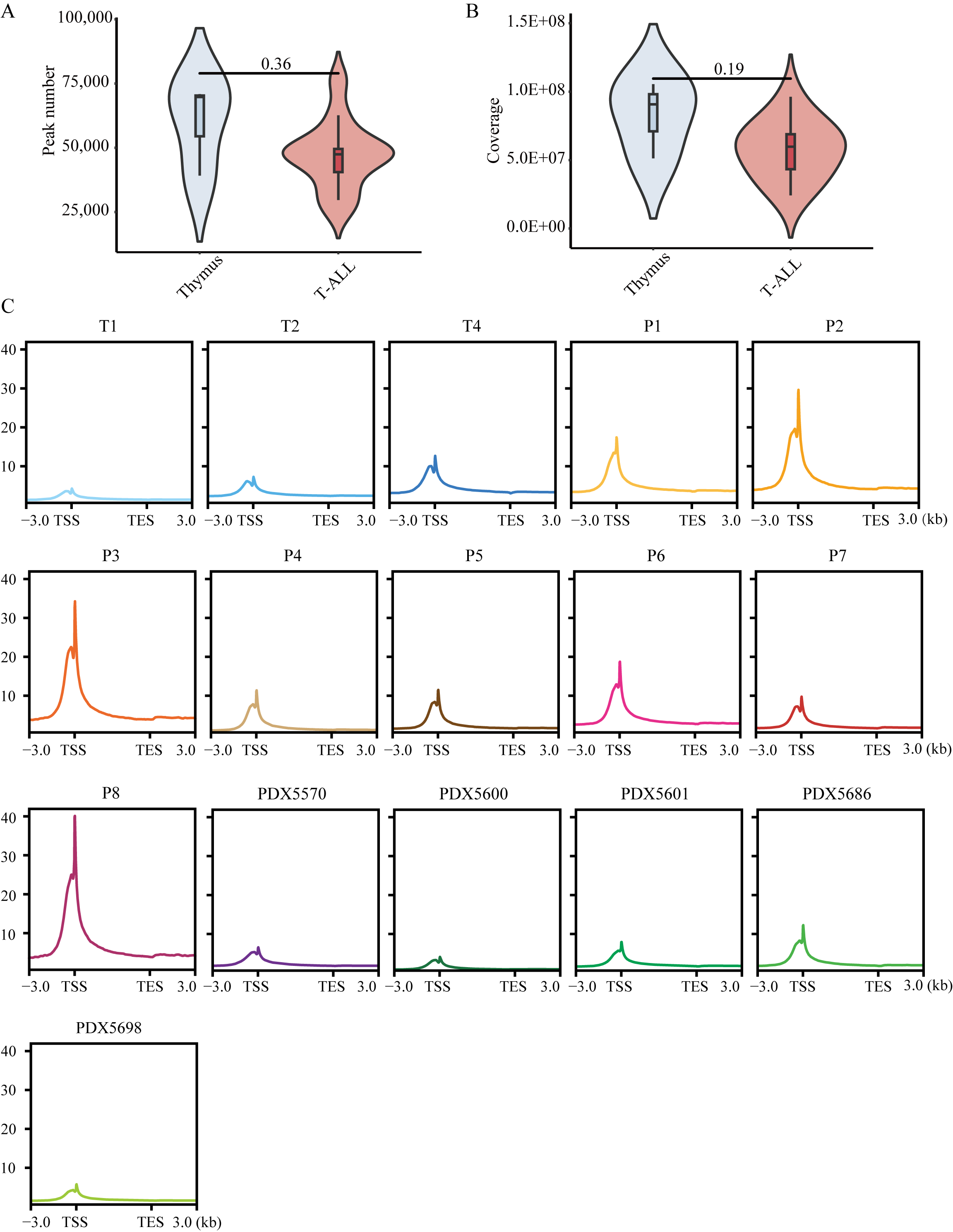

Supplement: qzaf029_Supplementary_Data [file qzaf029_supplementary_data.zip › Figure_S1.tif]

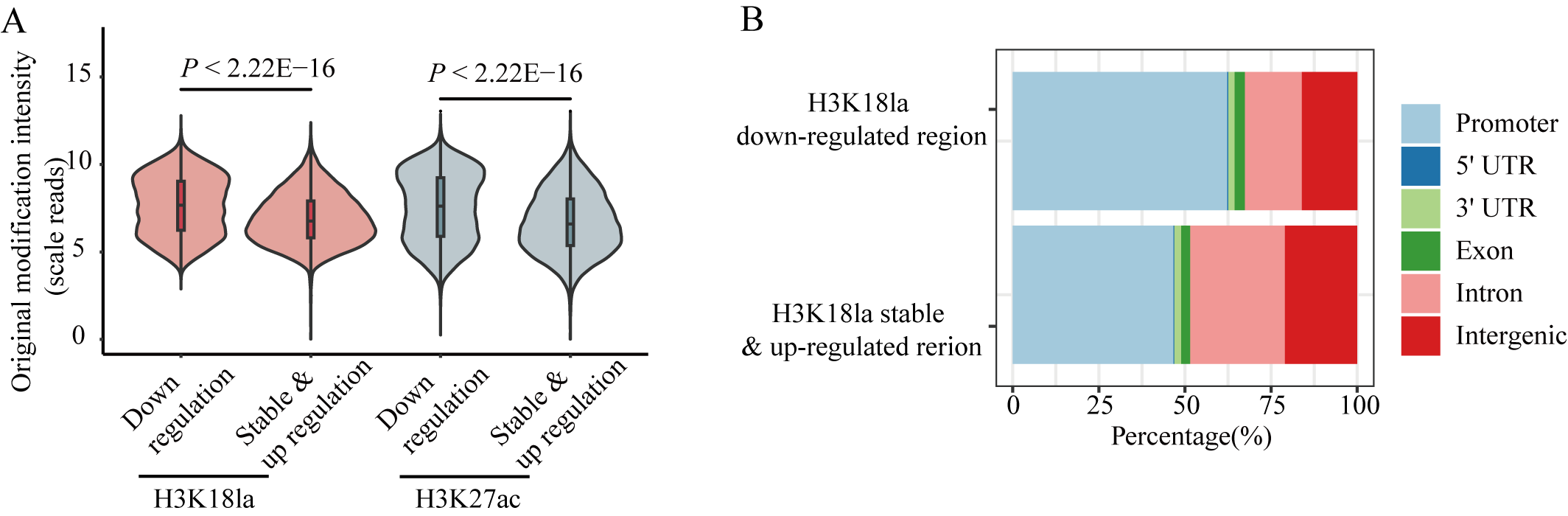

Supplement: qzaf029_Supplementary_Data [file qzaf029_supplementary_data.zip › Figure_S10.tif]

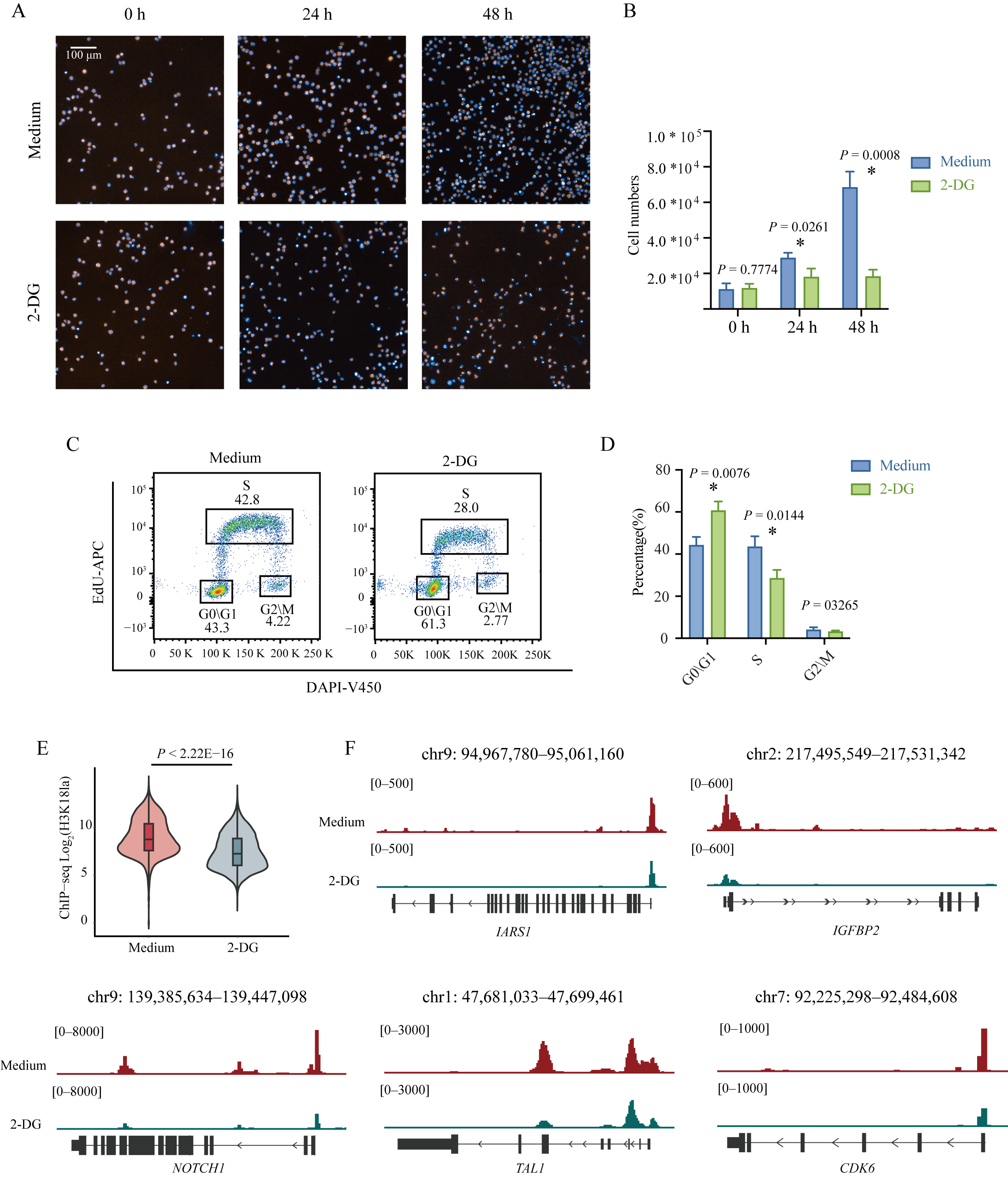

Supplement: qzaf029_Supplementary_Data [file qzaf029_supplementary_data.zip › Figure_S11.tif]

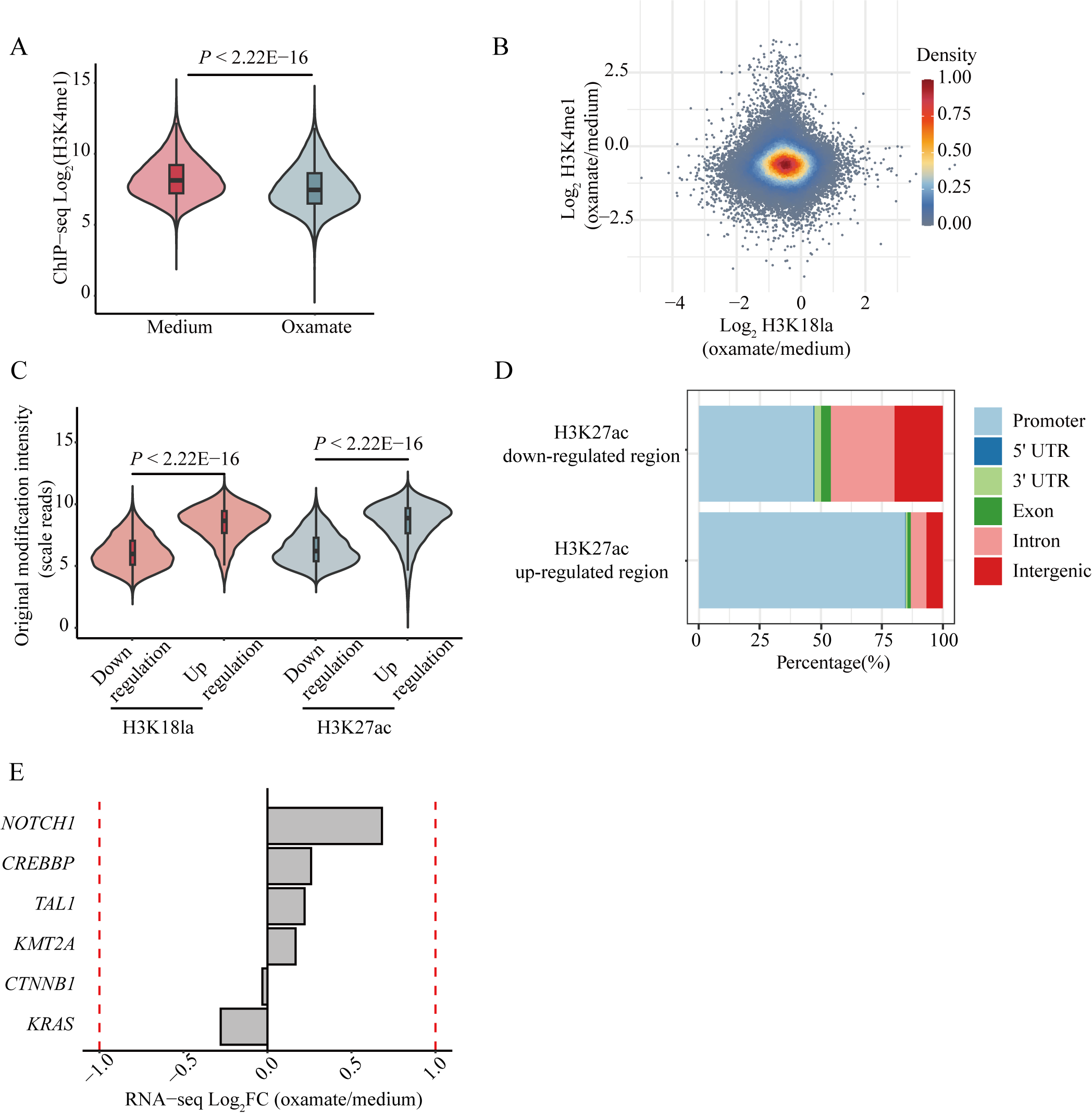

Supplement: qzaf029_Supplementary_Data [file qzaf029_supplementary_data.zip › Figure_S12.tif]

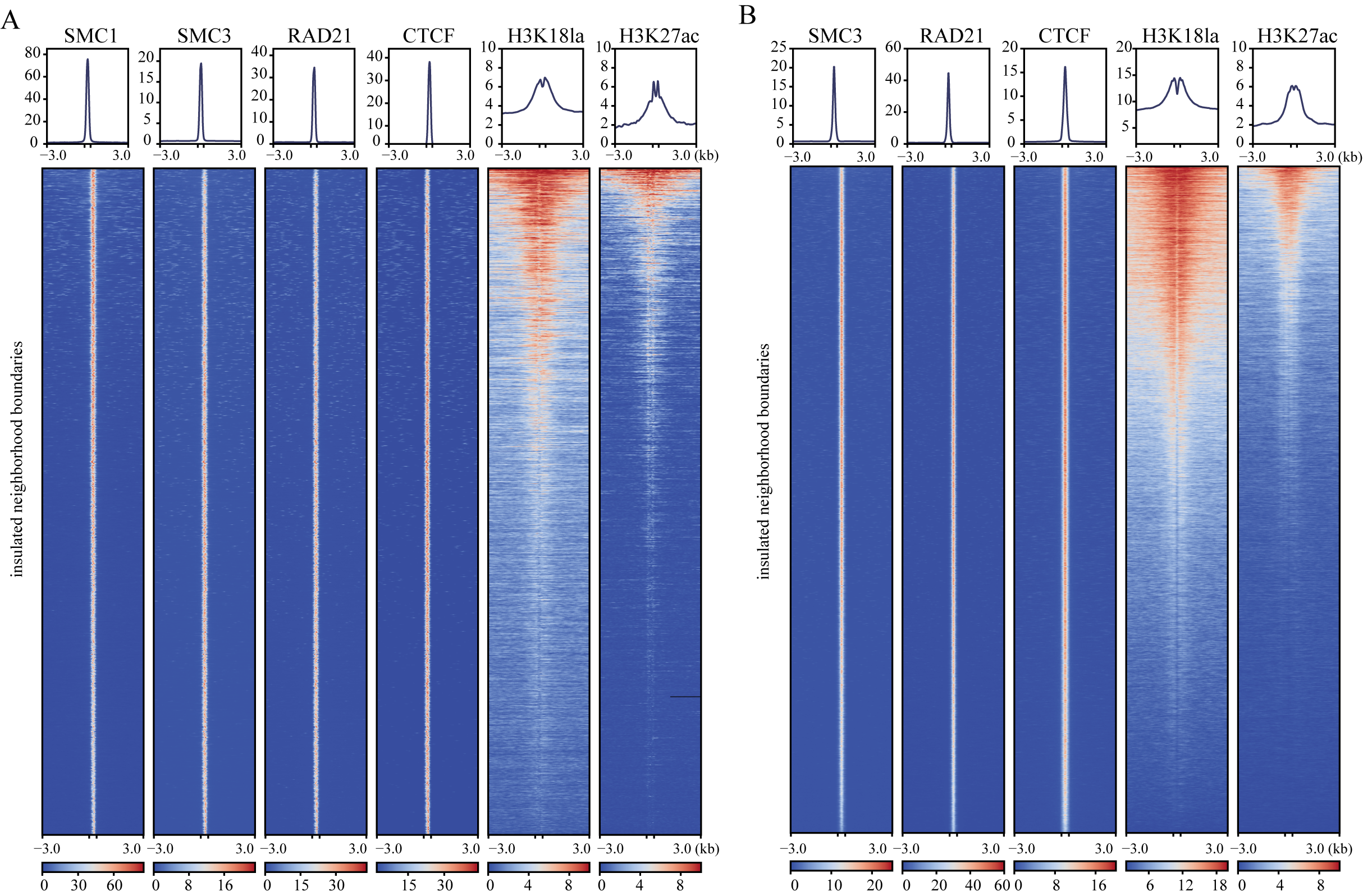

Supplement: qzaf029_Supplementary_Data [file qzaf029_supplementary_data.zip › Figure_S13.tif]

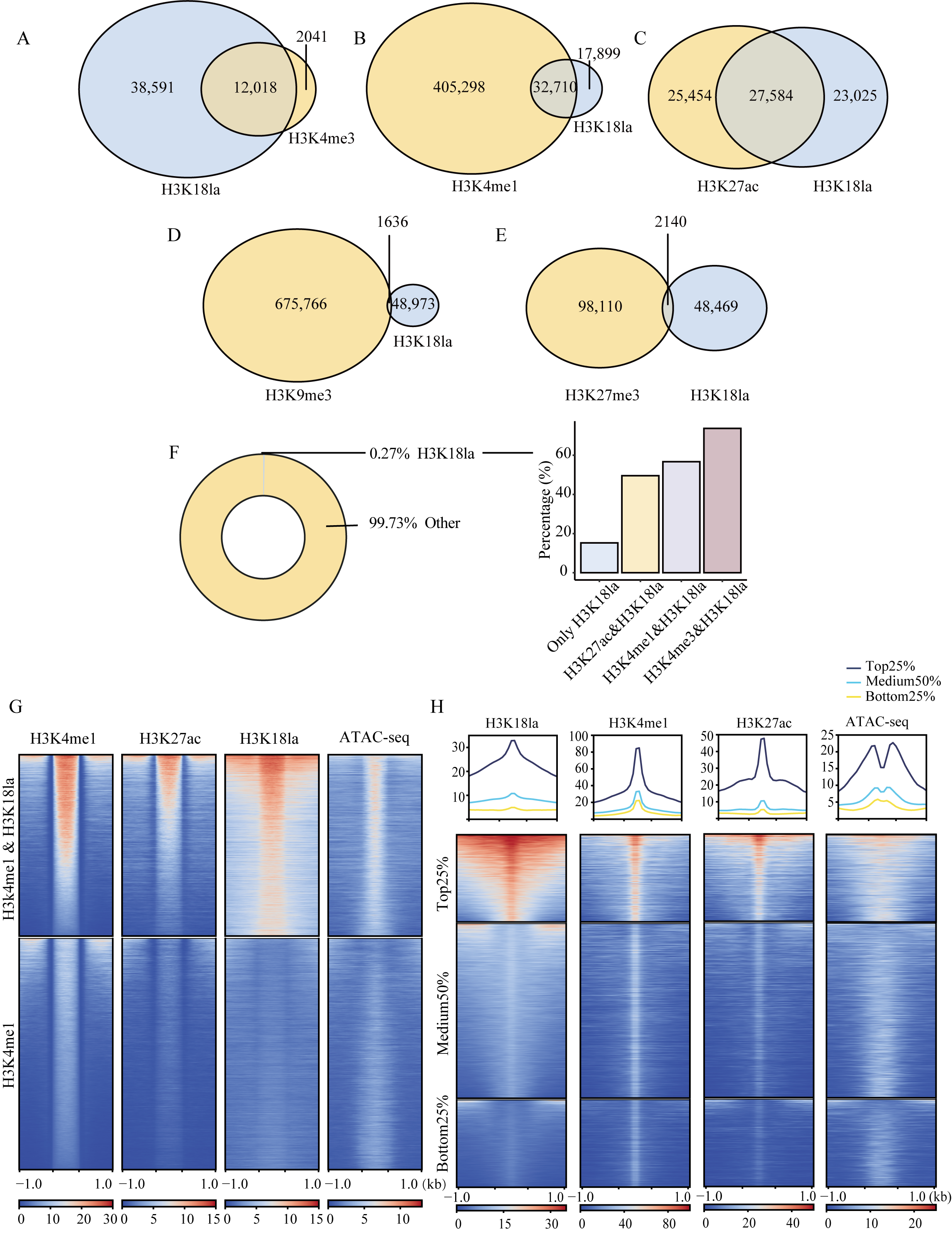

Supplement: qzaf029_Supplementary_Data [file qzaf029_supplementary_data.zip › Figure_S2.tif]

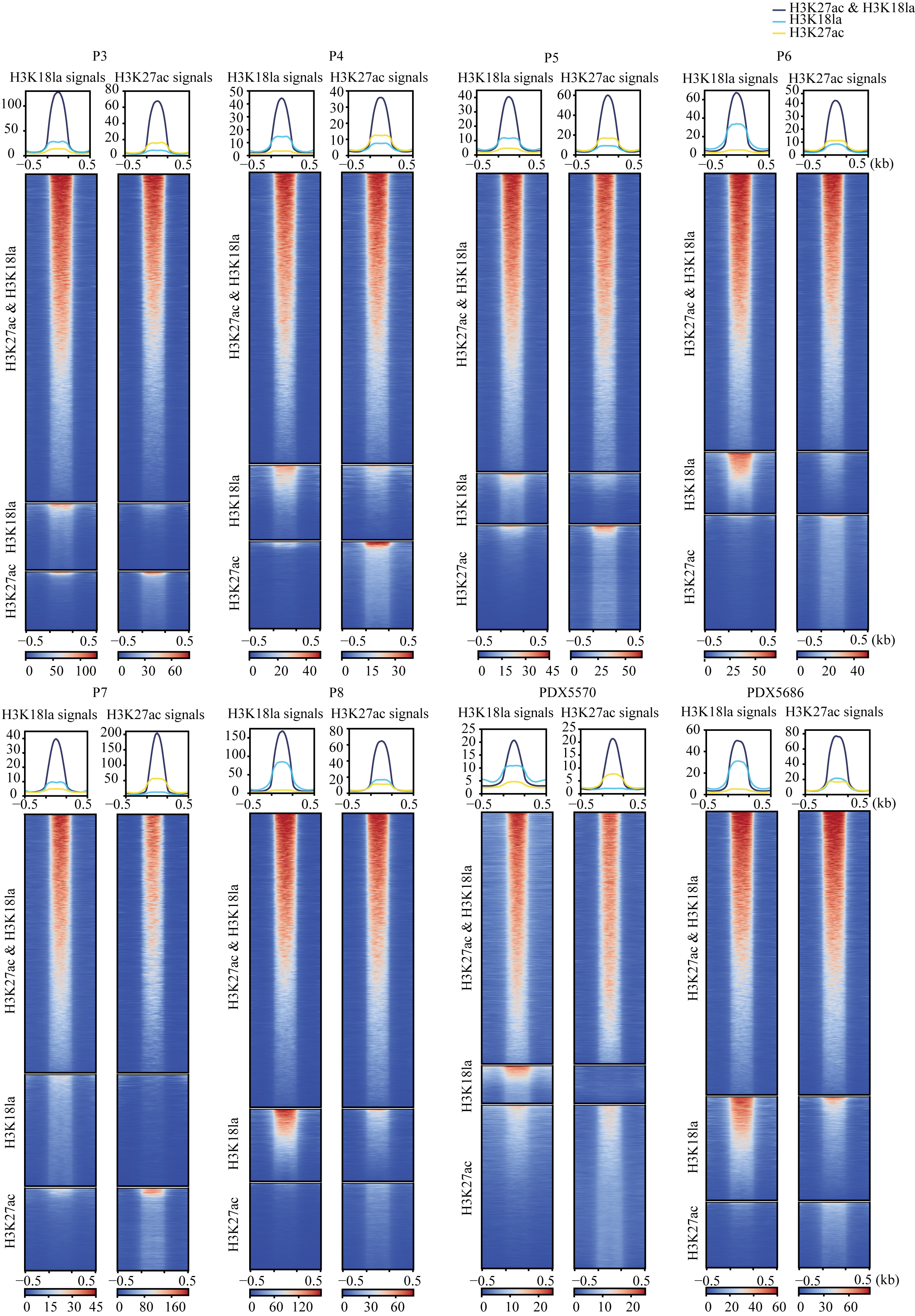

Supplement: qzaf029_Supplementary_Data [file qzaf029_supplementary_data.zip › Figure_S3.tif]

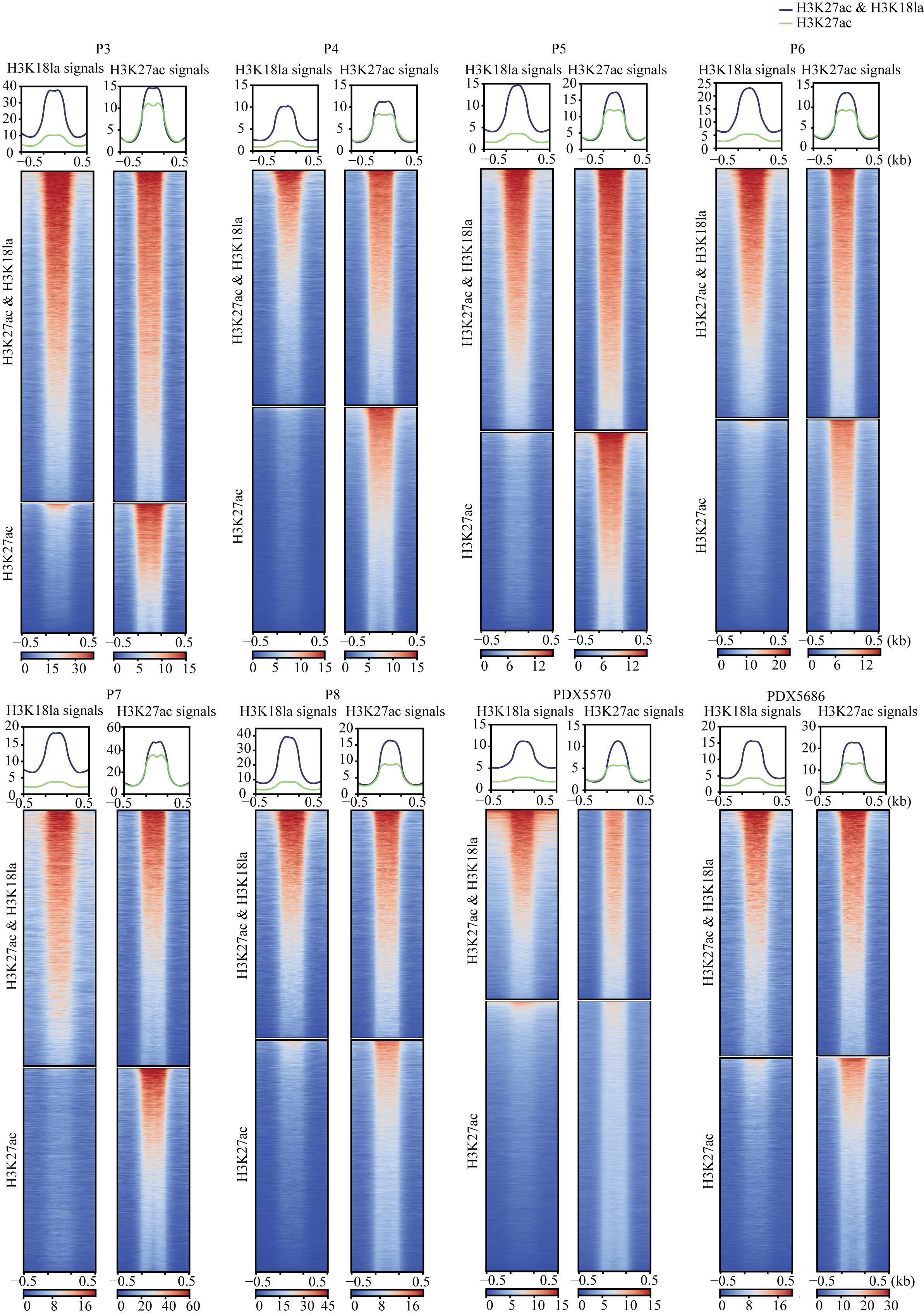

Supplement: qzaf029_Supplementary_Data [file qzaf029_supplementary_data.zip › Figure_S4.tif]

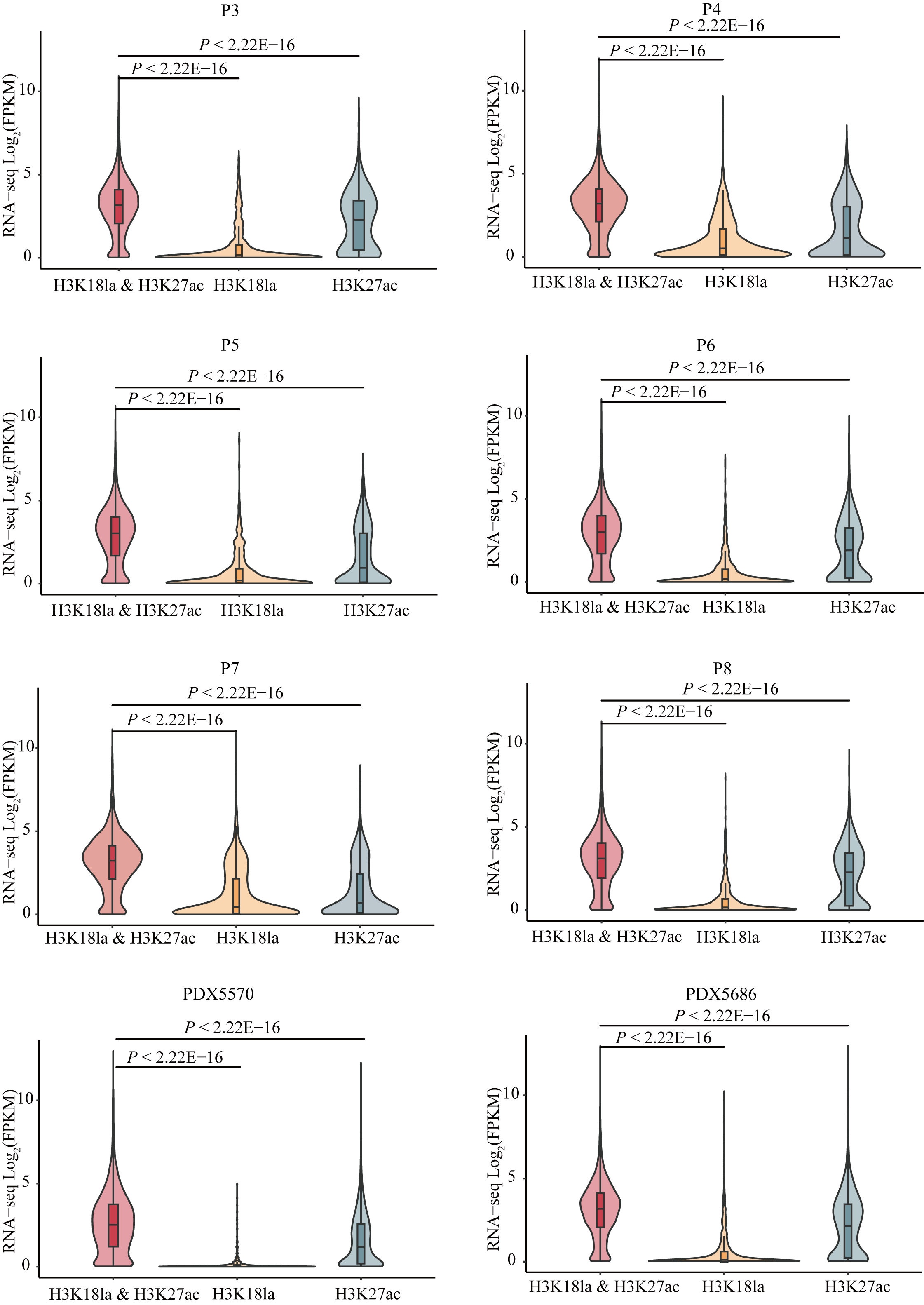

Supplement: qzaf029_Supplementary_Data [file qzaf029_supplementary_data.zip › Figure_S5.tif]

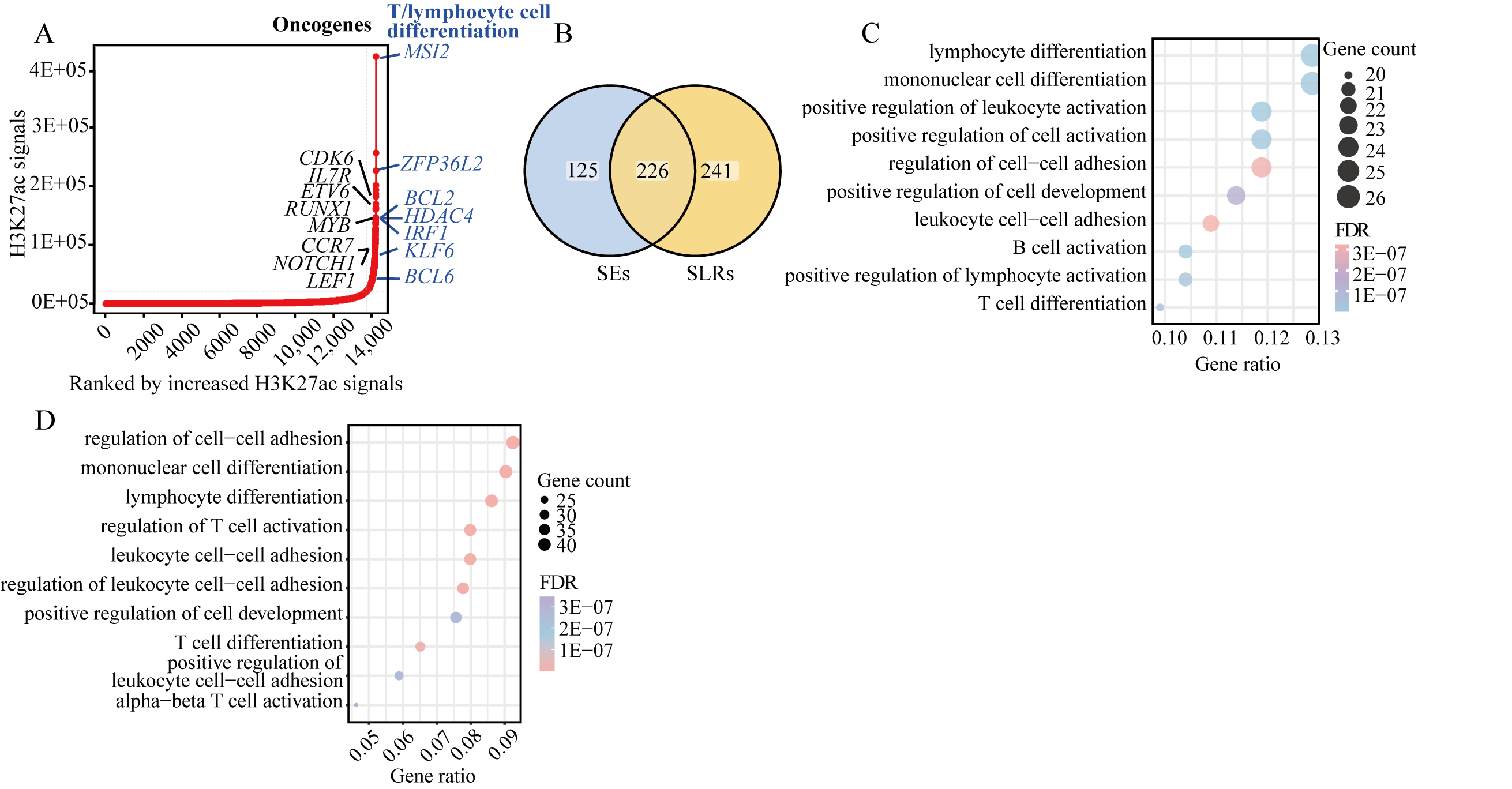

Supplement: qzaf029_Supplementary_Data [file qzaf029_supplementary_data.zip › Figure_S6.tif]

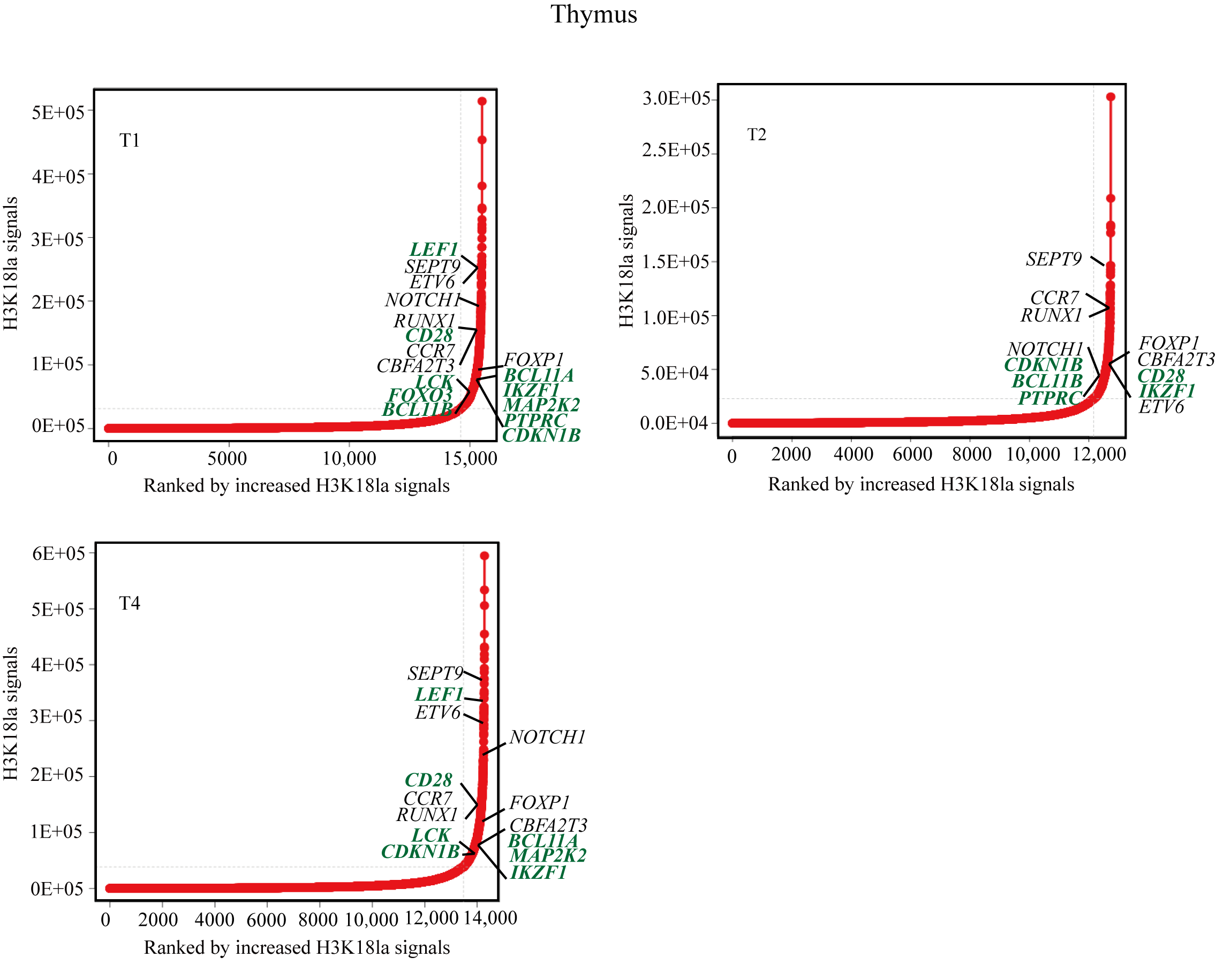

Supplement: qzaf029_Supplementary_Data [file qzaf029_supplementary_data.zip › Figure_S7.tif]

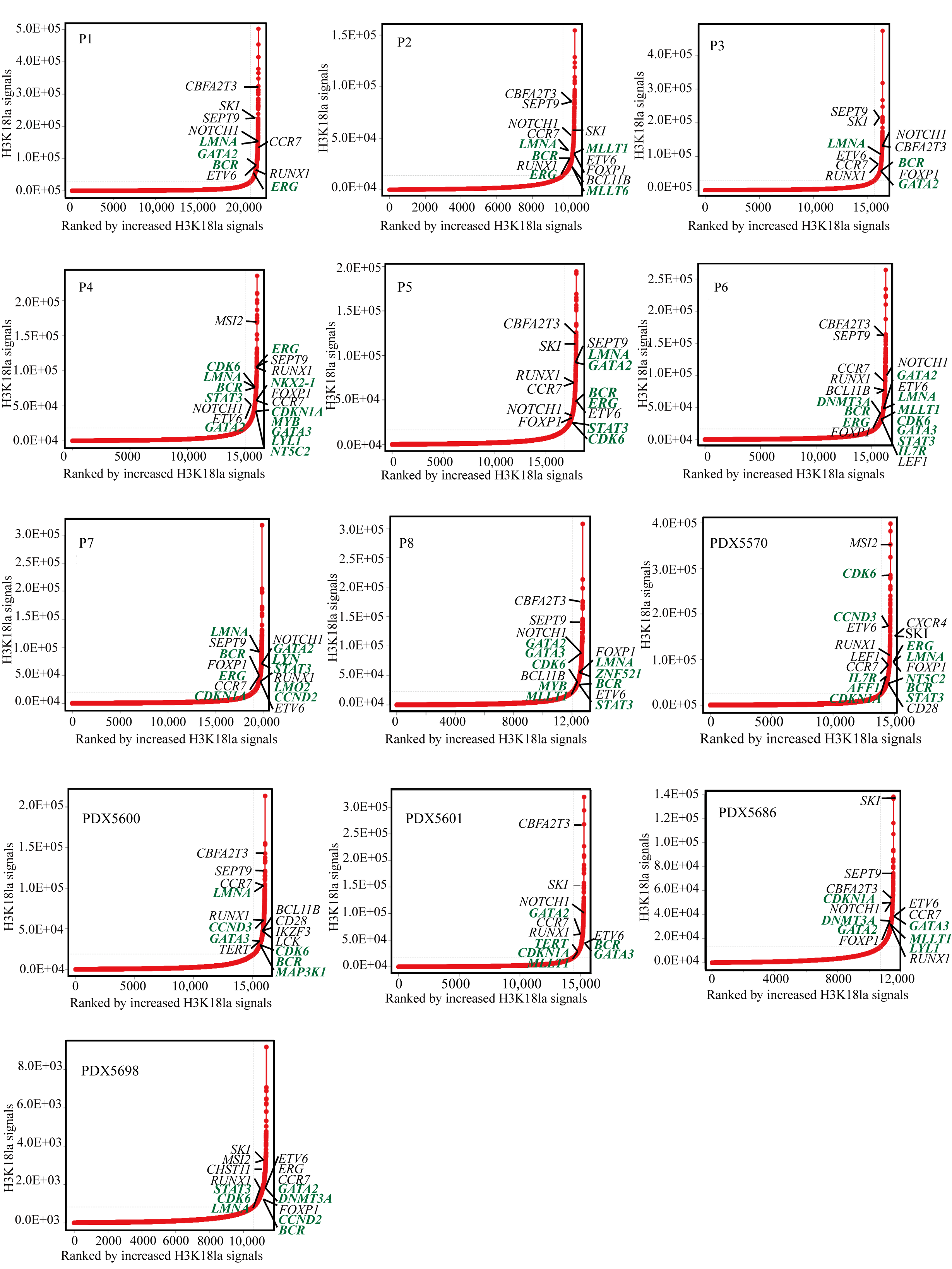

Supplement: qzaf029_Supplementary_Data [file qzaf029_supplementary_data.zip › Figure_S8.tif]

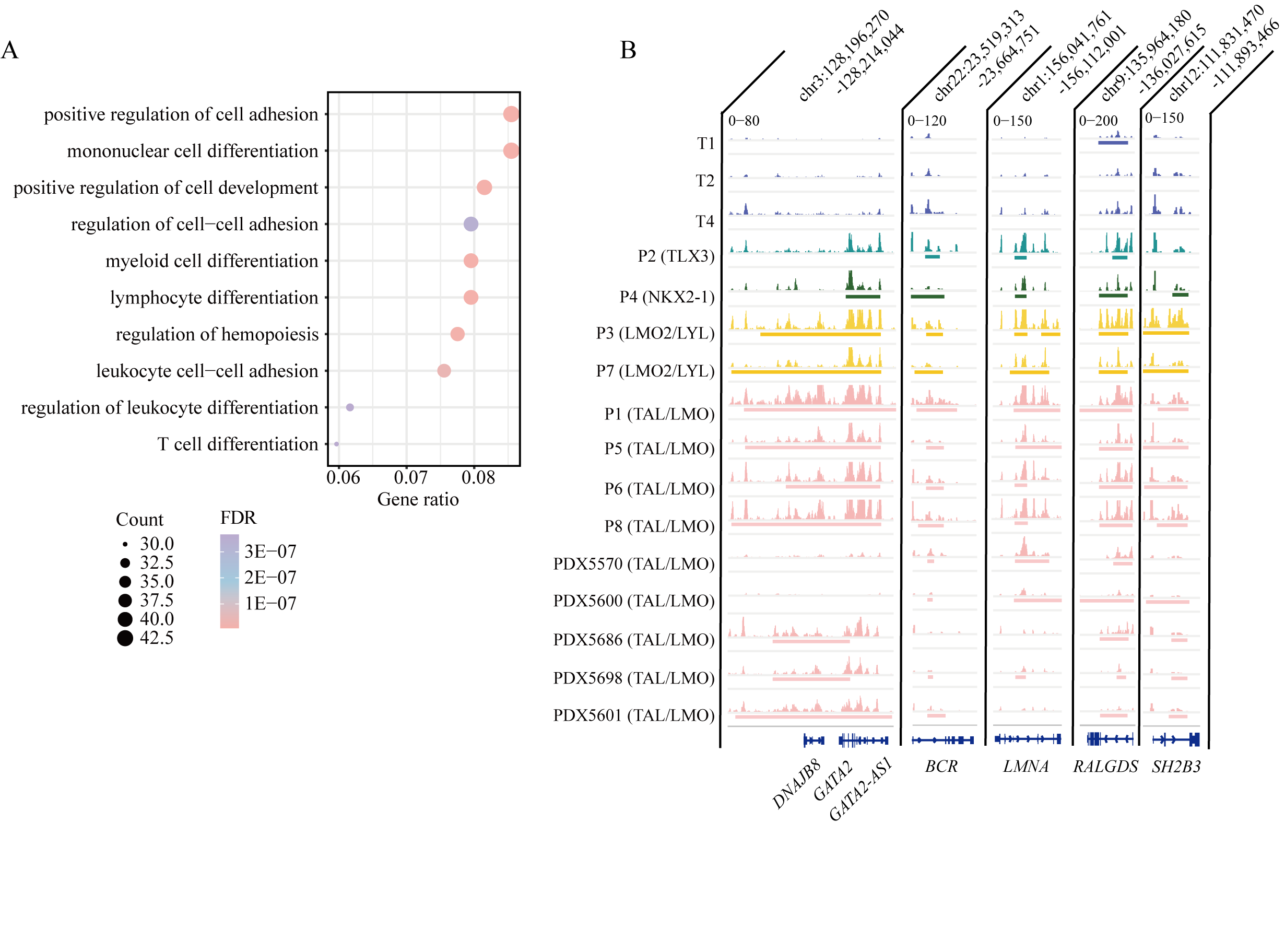

Supplement: qzaf029_Supplementary_Data [file qzaf029_supplementary_data.zip › Figure_S9.tif]
